# Administrative Claims Databases in Dentistry: Current Applications, Methodological Trends, and Evidence Gaps: A Global Scoping Review of Dataset Characteristics

**DOI:** 10.1155/ijod/8870016

**Published:** 2026-10-19

**Authors:** Misuzu Sato, Sachiko Ono, Miho Ishimaru, Yusuke Ida

**Affiliations:** ^1^ Department of Dental Public Health Faculty of Dental Medicine and Graduate School of Dental Medicine Hokkaido University Hokkaido 0608586 Japan hokudai.ac.jp; ^2^ Department of Eat-Loss Medicine Graduate School of Medicine The University of Tokyo 7-3-1 Hongo, Bunkyo-ku Tokyo 1130033 Japan u-tokyo.ac.jp; ^3^ Department of Dental Public Health Graduate School of Medical and Dental Sciences Institute of Science Tokyo 1-5-45, Yushima, Bunkyo-ku Tokyo 1138510 Japan tmd.ac.jp; ^4^ Department of Biomedical Informatics Graduate School of Medicine The University of Tokyo 7-3-1 Hongo, Bunkyo-ku Tokyo 1130033 Japan u-tokyo.ac.jp

**Keywords:** administrative claims database, dental epidemiology, dental research, healthcare utilization, policy impact, scoping review

## Abstract

**Objectives:**

Administrative claims databases are increasingly used in dental research. However, inconsistencies in data coverage and the lack of a global overview remain as challenges. This review aimed to summarize worldwide studies using dental administrative claims databases, focusing on research questions, methodologies, and findings to identify key trends and knowledge gaps.

**Methods:**

Following the Preferred Reporting Items for Systematic Reviews and Meta‐Analyses extension for Scoping Reviews (PRISMA‐ScR), we conducted a scoping review of studies using dental administrative claims data published between January 2010 and December 2025. We structured the review question using the Population‐Concept‐Context framework. The original search through April 2022, as registered in PROSPERO, was updated in April 2026 using the same search strategy and eligibility criteria. We searched PubMed, Ovid MEDLINE, Ichushi‐Web, CiNii, Scopus, and Web of Science databases. Two reviewers independently screened the records, and disagreements were resolved by consensus. Cohort and case–control studies were critically appraised using the Newcastle–Ottawa Scale (NOS) to characterize the methodological quality of the mapped literature.

**Results:**

Following screening, 472 studies met the eligibility criteria. Most studies originated from the United States and Taiwan, indicating marked geographical concentration; the updated search added Switzerland and New Zealand. Research topics included treatment effectiveness, the epidemiology of rare conditions, healthcare utilization, and policy evaluation. Common limitations were insufficient clinical detail, variable data quality, and limited generalizability.

**Conclusions:**

This review highlights the expanding use of administrative claims databases in dental research and their inherent limitations. Future research should prioritize validating claims‐based diagnosis and procedure codes and linking claims data with clinical records.

## 1. Introduction

The use of administrative claims databases in dental research has markedly increased. Initially compiled for billing and administrative purposes, these databases provide extensive data that enable large‐scale, population‐based studies [1–3]. Studies using administrative claims databases cover the epidemiology of dental diseases [4], assessment of treatment outcomes [5], evaluation of healthcare utilization and costs [6], and analysis of policy impacts [7].

Despite the existence of administrative claims databases in some countries, dental health insurance coverage varies substantially across regions in terms of population, services, and costs [8]. Claims data also lack detailed clinical information. Additionally, the information extracted from these databases varies widely; some countries include detailed information on oral health, such as the number of teeth, by linking dental checkup data, whereas others include only procedure and medication data. Studies offering a comprehensive overview of global research using claims data in dentistry, detailing what information is combined and which topics are investigated, remain scarce.

This scoping review aimed to map the extent, range, and nature of research using dental administrative claims databases, with specific emphasis on the countries and data sources represented, types of research questions addressed, study designs and analytical methods applied, and limitations reported. The review aimed to identify patterns and gaps in the literature, offering insights into the strengths and limitations of using administrative claims data in dental research.

## 2. Methods

We conducted a scoping review following the Preferred Reporting Items for Systematic Reviews and Meta‐Analyses extension for Scoping Reviews (PRISMA‐ScR) [9] and the methodological guidance of the Joanna Briggs Institute [10]. A scoping review framework was selected because the objective was to map the extent, range, and nature of a heterogeneous literature rather than answer a focused clinical or epidemiological question or synthesize effect estimates across studies. Under these circumstances, a scoping review, rather than a systematic review, is the appropriate approach [11, 12]. The protocol was registered in PROSPERO as a systematic review (registration number: CRD42022338592); reframing the review as a scoping review and extending the search end date (Section 2.1) are the two deviations from the registered protocol. Our review addressed the following questions: (1) How many studies in dentistry have used administrative claims databases? (2) What types of studies have been conducted? (3) What are the strengths and limitations of these studies?

Consistent with the guidance for scoping reviews, these questions were structured using the Population‐Concept‐Context framework. The population comprised published reports of original research conducted using administrative health insurance claims databases containing dental or oral health records. Therefore, the unit of analysis was a published article rather than a group of individuals. The concept included the distribution of study designs, statistical and analytical methods, clinical application areas, and research topics addressed in these reports, together with limitations reported by the authors. The context included the country or region in which each study was conducted, the database used, whether the database was national or regional in scope, and the calendar period covered. The eligibility criteria set out in Section 2.1 were operationalized against these three elements.

### 2.1. Information Sources, Search Strategy, and Study Selection

We searched PubMed, Ichushi‐Web (Japan Medical Abstracts Society), CiNii, MEDLINE, Scopus, and Web of Science databases between January 2010 and April 2022. MEDLINE was searched using the Ovid platform (Ovid MEDLINE). Although PubMed indexes MEDLINE records, retrieval through the two interfaces was not identical; therefore, both were searched. Records from all sources were combined and deduplicated in Rayyan (Rayyan Systems, Inc.) before screening. The number of duplicate records removed at each stage is reported in Figure 1. To ensure currency, the original search was rerun using the same strategy across all databases, extending coverage through December 2025 (last searched in April 2026). Newly identified records were screened using the same eligibility criteria by two independent reviewers; 159 additional studies met the inclusion criteria, and the PRISMA flow diagram, study characteristics tables, and synthesis were updated accordingly. The searches were conducted on May 30–31, 2022, and April 21, 2026, using terms related to administrative claims databases and dentistry. The full PubMed search strategy is presented in Table 1. The search terms for PubMed (in English) and Ichushi‐Web (in Japanese) were developed by the authors and librarians from the Medical Library at the University of Tokyo. These terms were then adapted to the other databases. Search terms were deliberately generic, denoting claims, administrative, or health insurance data, rather than enumerating proprietary database names, such as MarketScan, the National Health Insurance (NHI) Research Database, HCUP, Optum, TriNetX, or SEER‐Medicare. The number of administrative claims databases worldwide is large and cannot be specified in advance. Naming a subset of data sources would have biased the retrieval toward the small number of countries with internationally recognized databases, precisely the geographical concentration this review set out to describe. The reference lists of the included studies were also manually searched.

**Figure 1:**
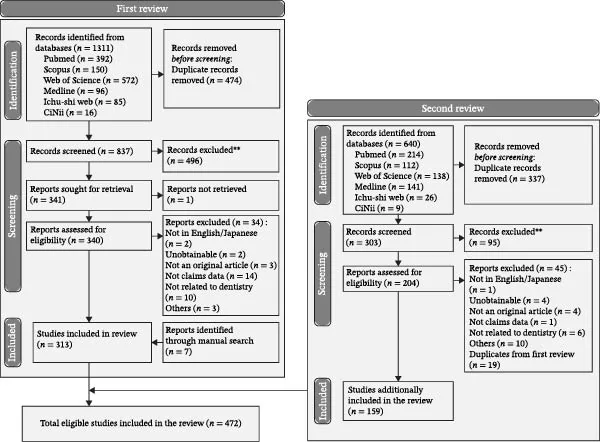
Flow diagram of the study selection process.

**Table 1:** Search strategy for the primary database (PubMed).

((“administrative claims, healthcare”[MeSH Terms] OR “claim data”[Title/Abstract] OR “claims data”[Title/Abstract] OR “claim database”[Title/Abstract] OR “claims database”[Title/Abstract] OR “receipt database”[Title/Abstract] OR “administrative data”[Title/Abstract] OR “administrative database”[Title/Abstract] OR “insurance data”[Title/Abstract] OR “insurance database”[Title/Abstract] OR “Medicare database”[Title/Abstract] OR “Medicaid database”[Title/Abstract] OR “Medicare data”[Title/Abstract] OR “Medicaid data”[Title/Abstract] OR “Insurance, Health”[Title/Abstract]) AND (“dentistry”[MeSH Terms] OR “Jaw Diseases”[MeSH Terms] OR “Mouth Diseases”[MeSH Terms] OR “Tooth Diseases”[MeSH Terms] OR “oral health”[MeSH Terms] OR “dental Health Services”[MeSH Terms] OR “dental facilities”[MeSH Terms] OR “dental informatics”[MeSH Terms] OR “oral disease”[Title/Abstract] OR “oral diseases”[Title/Abstract] OR “oral health”[Title/Abstract] OR “dental health”[Title/Abstract])) AND 2010/01/01: [2025/12/31][dp]

Eligibility was defined by the data source rather than by the design label. Cohort, case–control, cross‐sectional, descriptive, ecological, interrupted time‐series, data‐based simulation, and code‐validation studies were all eligible. Simulation studies were eligible only when they were parameterized with and applied to empirical claims data; purely theoretical simulation studies without claims data were excluded. All records identified from the database searches were managed in Rayyan, with duplicates removed. Two reviewers independently screened the titles and abstracts to exclude studies based on the following criteria: (1) nonoriginal articles, (2) articles not using an administrative claims database, (3) articles published in languages other than English or Japanese, and (4) articles unrelated to dentistry or oral health. Discrepancies were resolved by consensus with a third reviewer. Four reviewers then assessed the full texts, and disagreements were resolved by consensus.

### 2.2. Data Extraction

We extracted the following data from each included study: (1) publication year, (2) title and authors, (3) country, (4) geographical scope (national or regional, with region where available), (5) database characteristics (described below), (6) study design, (7) exposure and control, (8) outcomes, (9) statistical methods, (10) limitations, and (11) clinical application (described below).

A predefined charting form was consistently used to collect information in three review domains: database characteristics, study design and statistical methods, and clinical application.

#### 2.2.1. Database Characteristics

We charted the geographic scope (national or regional, with region names where applicable), the primary database, and any linked or merged databases.

#### 2.2.2. Study Design and Statistical Methods

Each study was classified as either descriptive or analytical. Analytical studies were further categorized as ecological, cross‐sectional, case–control, or cohort. A second design field included studies that combined multiple designs. Statistical methods were recorded verbatim as free text. Study limitations were coded using a predefined scheme (up to four coded categories plus free text) to capture issues such as misclassification or measurement error in exposure or outcome, limited availability of laboratory or clinical parameters, and generalizability.

#### 2.2.3. Clinical Application

Studies were tagged using a multilabel scheme rather than a single primary category. One set of tags described the dental clinical topic: periodontitis, dental caries or preventive care, prosthesis, oral and maxillofacial surgery, dental visit or procedure, health policy or program, dental cost, and other. A second set described linked systemic conditions (e.g., diabetes, dementia, cancer, cardiovascular disease, cerebrovascular disease, respiratory disease, kidney disease, osteoporosis, rheumatoid arthritis, allergic rhinitis, pregnancy, and osteonecrosis of the jaw), together with a tag for overall healthcare cost. A study could receive multiple tags across both sets.

Categories for the clinical topic and systemic condition tags were not defined a priori. No established taxonomy existed for this literature, and categories were therefore developed inductively during charting. For each included article, the principal exposure and principal outcome were identified from the full text, and the articles were grouped according to the clinical topic of those variables. Separate categories were retained for topics with enough studies to be informative; topics with few studies were assigned to “other.” Charting was performed by one reviewer and checked against the full texts by the remaining authors. Queries were resolved by discussion, and no unresolved disagreements arose.

### 2.3. Critical Appraisal

Consistent with the scoping review methodology, a critical appraisal was undertaken to characterize the methodological quality of the mapped literature, not to weight, pool, or exclude studies. We appraised cohort and case–control studies using the Newcastle–Ottawa Scale (NOS). The NOS is a star‐based instrument comprising three domains. In the cohort version, the selection domain comprises four items: representativeness of the exposed cohort, selection of the nonexposed cohort, ascertainment of exposure, and demonstration that the outcome of interest is not present at the start of the study; each is awarded a maximum of one star. The comparability domain comprises a single item awarded a maximum of two stars: one for controlling the principal confounder and one for controlling any additional confounder. The outcome domain comprises three items: assessment of outcome, adequacy of the duration of follow‐up, and adequacy of the completeness of follow‐up; each is awarded a maximum of one star. In the case–control version, the selection and comparability domains are scored equivalently, and the outcome domain is replaced with an exposure domain comprising three items: ascertainment of exposure, use of the same ascertainment method for cases and controls, and nonresponse rate. Therefore, the maximum attainable score is four stars for selection, two for comparability, and three for outcome or exposure, giving nine stars in total for both appraised designs.

We classified the overall risk of bias as low for total scores of seven to nine stars, moderate for those of four to six stars, and high for those of three stars or fewer. The domain‐level risk was classified as low when a domain was awarded its full complement of stars, moderate when one star was missing, and high when two or more stars were missing. Therefore, selection was rated low at four stars, comparability at two stars, and outcome or exposure at three stars. The domain‐level and total scores for each appraised study are reported in Table S5.

The study designs to which the instrument was not applicable, including cross‐sectional, descriptive, ecological, interrupted time‐series, simulation, and validation studies, were not appraised. The number of studies in this category is reported in Section 3.5. A first‐pass, item‐level draft rating was generated using a large language model, ChatGPT, Model GPT‐5.5 Thinking (OpenAI, San Francisco, CA, USA; accessed via https://chatgpt.com/g/g-Xr35IAPKg-newcastle-ottawa-quality-assessment-scale-nos) between June and July 2026. The full text of each eligible article was supplied to the model together with a standardized prompt requesting a rating for every NOS item and the sentence from the article supporting that rating. The prompt is reproduced verbatim in Appendix 1. Every model‐generated item rating was then checked against the source article by one reviewer who read the relevant sections of the full text and revised any rating that was not supported by the article. Therefore, the ratings reported here are reviewer‐determined. The model was used only to produce a draft for verification, and no model output was accepted as a final judgment. As every rating was independently verified, the agreement between the model‐generated and reviewer‐determined ratings was not treated as a measure of validity and was not reported.

We did not assess the publication bias or the certainty of the body of evidence. Both assessments presuppose a set of studies addressing a common question, whose results are pooled into a synthesized effect estimate. This review maps the characteristics of the heterogeneous literature and does not synthesize outcome data across studies; therefore, there is no common estimate against which small‐study effects or certainty of evidence can be judged.

## 3. Results

### 3.1. Search Results

The flow diagram of the study selection process is shown in Figure 1. The initial search yielded 1311 articles from the selected databases. After removing duplicates, 837 unique records were identified. After screening titles and abstracts according to the eligibility criteria, 496 articles were excluded. Of the remaining 341 reports sought for retrieval, one could not be obtained; the full texts of 340 articles were assessed for eligibility, leading to the exclusion of 34 additional articles, leaving 306 eligible studies. Seven additional reports were identified through a manual search, resulting in 313 studies from the original review. The updated search identified a further 640 records, of which 337 duplicates were removed, leaving 303 records for screening. Of these, 99 were excluded based on the title and abstract, and 204 were assessed for eligibility; 45 were excluded, including 19 studies already included in the first review, leaving 159 additional eligible studies. Finally, 472 articles were included in this scoping review.

### 3.2. Characteristics of Included Studies

All the included studies are listed by country in Table S1. The studies included in this scoping review were conducted between 2010 and 2025. Figure 2 shows an increasing trend in the number of studies using administrative claims databases in dental research. The annual number of studies remained at 40 or more from 2021, reaching 49 by 2025. Notably, NOS‐eligible studies accounted for approximately half of all publications in recent years.

**Figure 2:**
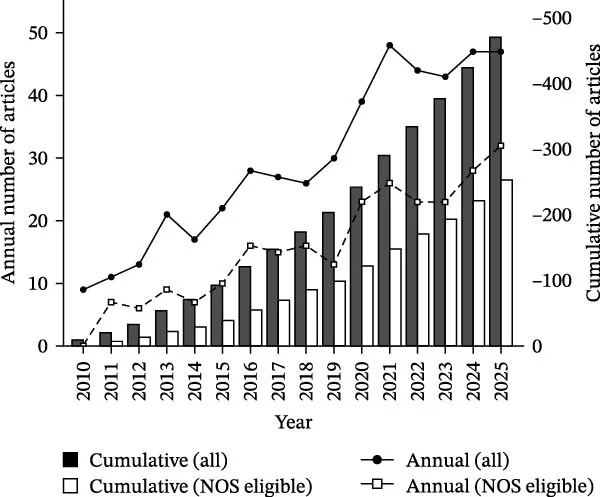
The number of studies using administrative claims databases in dental research. *Note:* The solid line represents the cumulative number of studies, whereas the dashed line indicates the annual number of studies. Data extend through 2025.

Table 2 lists the countries and databases of all included studies. The country with the highest number of studies (175 studies) was the United States, drawing on a range of public and commercial datasets. Taiwan contributed 93 studies, primarily using the NHI Research Database. Japan contributed 75 studies, mainly using the National Database of Health Insurance Claims and Specific Health Checkups of Japan (NDB) and the Kokuho Database (KDB). South Korea (39), Germany (22), the United Kingdom (22), France (11), Canada (11), the Netherlands (7), Australia (6), Denmark (4), Switzerland (3), Austria (1), Finland (1), Italy (1), and New Zealand (1) contributed fewer studies. The updated search added two countries not previously represented (Switzerland and New Zealand), bringing the total number of contributing countries to 16. This distribution highlights the concentration of dental claims database research in countries with established health data systems. Studies in countries such as Taiwan, South Korea, Germany, France, and the Netherlands primarily utilized national databases, whereas those in the United States, Japan, and the United Kingdom used both national and regional databases. The studies in the United States and Germany made greater use of commercial databases.

**Table 2:** Countries and databases of all included studies.

Country (*n*)	Database	National/regional	Oldest research
United States (175)	Aetna Inc.	National	2009
	All‐Payer Claims Database	Regional	2010
	Caris Life Sciences molecular profiling	National	2024
	Centers for Medicare and Medicaid Services (CMS) data	National/regional	1995
	Community dental access program (CDP)	Regional	2011
	Consumer directed health plan (CDHP)	National	2012
	Delta Dental claims data	Regional	2002
	Dental insurance claims data (New York State and Massachusetts)	Regional	2023
	Express Scripts Members	National	2013
	HealthPartners insurance data	Regional	2023
	Healthcare Cost and Utilization Project (HCUP)	National	2000
	Hospital‐based database	Regional	2010
	IBM MarketScan databases	National/regional; national	2006
	Medicaid/CHIP claims data	Regional; national	1998
	Medicare claims data	Regional	2025
	National Surgical Quality Improvement Program (NSQIP)	Regional	2009
	OMS National Insurance Company (OMSNIC) malpractice claims	National	2022
	Optum Clinformatics Data Mart	National	2001
	P&R Dental Strategies/Fluent dental claims warehouse	National	2023
	PearlDiver database	Regional	2023
	SEER Program	National	1998
	TRICARE health record data	National	2010
	Transformed Medicaid Statistical Information System (T‐MSIS)	Regional; national	2022
	TriNetX claims data	National	2025
	Washington State dental/Medicaid claims data	Regional	2002
	Wolters kluwer dynamic claims lifecycle database	National	2007
Taiwan (93)	Longitudinal Health Insurance Database (LHID/LHIRD)	National	1996
	Taiwan National Health Insurance Research Database (NHIRD/NHI)	National	1996
Japan (75)	DeSC health insurance claims database	Regional	2024
	Hospital‐based database	Regional	2012
	JMDC Claims Database	National; regional	2014
	Japan Gerontological Evaluation Study (JAGES)	National	2025
	Kokuho Database (KDB/SKDB)	Regional	1997
	Late Elders’ Health Insurance database	Regional	2025
	Longevity Improvement and Fair Evidence (LIFE) Study	National/regional	2023
	Medical Data Vision (MDV) database	Regional	2025
	MinaCare claims database	Regional	2025
	National Database of Health Insurance Claims and Specific Health Checkups of Japan (NDB)	National; national/regional	2011
	National health checkup program data (Japan)	Regional	2024
	Oral Health Screening to Assess Keys of Aging Well (OHSAKA) study	Regional	2025
	Other Japanese health insurance, claims, or checkup databases	National/regional; regional; national	2002
South Korea (39)	Health Insurance Review and Assessment (HIRA) database	National	2002
	Korean National Health Insurance Service database (NHIS/KNHIS)	National	2002
	NHIS National Health Screening Cohort (NHIS‐HEALS)	National	2002
	NHIS National Sample Cohort	National	2022
Germany (22)	AOK health insurance claims data	National; regional	2012
	BARMER health insurance claims data	National	2004
	BFS health finance claims data	National/regional	2024
	German statutory health insurance claims data	National; regional	2013
	InGef research database	National	2011
United Kingdom (22)	Cleft Registry and Audit NEtwork (CRANE)	National	2023
	Dental Treatment Benefit Scheme/HSE data	National; regional	1996
	Hospital Episode Statistics/HED	National; regional	1997
	NHS Scotland administrative data	Regional	2023
	NHS dental claims and administrative data	Regional; national	1990
Canada (11)	Alberta administrative/registry data	Regional; national	2024
	CIHI Discharge Abstract Database	Regional	1994
	Noninsured Health Benefits (NIHB) claims data	National	2025
	Ontario administrative/dental public health data	Regional	2011
	The medical claims/physician billings database	Regional	1997
France (11)	ALD long‐term disease registry	National	2000
	E3N cohort	National	2011
	French National Health Insurance/SNIIRAM‐SNDS data	National	2008
	Health and Social Protection Survey (ESPS)	National	2006
Netherlands (7)	Achmea/Zilveren Kruis health insurance claims data	National	2000
	Dental practice‐based research network data	National	—
	Dutch health insurance claims data	Regional	2022
Australia (6)	Department of Veterans’ Affairs (DVA) data	National	2016
	Medical Benefits Fund (MBF) data	National	2007
	Medicare/Child Dental Benefits Schedule (CDBS) data	National	2011
	Queensland Health administrative dental data	Regional	2025
Denmark (4)	Danish Dental Compensation Act (DDCA) database	National	2024
	Danish National Health Insurance database	National	2009
	The Copenhagen Primary Care Laboratory (CopLab) database	National	2000
Switzerland (3)	Concordia (Swiss health insurer) accident claims data	National	2024
	National Inpatient Sample	National	2024
	Swiss sports injury insurance claims database	National	2023
Austria (1)	Population‐based health insurance data	National	2012
Finland (1)	The Social Insurance Institute database of private dental care services (aggregated)	National	2012
Italy (1)	Bisphosphonates Effectiveness Safety Trade‐off (BEST) network	Regional	2003
New Zealand (1)	National administrative health data (New Zealand)	National	2025

*Note:* Databases with variant names across studies were consolidated into standardized database groups. The per‐study reference IDs and original database names are listed in Table S2.

Table 3 summarizes the study designs. Cohort studies were predominant (*n* = 226; 47.9%), followed by cross‐sectional studies (*n* = 109; 23.1%), descriptive analyses (*n* = 77; 16.3%), and case–control studies (*n* = 36; 7.6%). Interrupted time‐series, simulation, and validation designs were rarely used.

**Table 3:** Study designs of included studies.

Study design	*n* (%)
Cohort study	226 (47.9)
Cross‐sectional study	109 (23.1)
Descriptive analysis	77 (16.3)
Case–control study	36 (7.6)
Ecological study	8 (1.7)
Interrupted time‐series study	4 (0.8)
Validation study	4 (0.8)
Simulation study	1 (0.2)
Others^a^	7 (1.5)

^a^Others include publications that are difficult to categorize, such as those using original research designs or not clearly indicating their research design. Reference IDs are listed in Table S3.

### 3.3. Exposure and Outcome Variables

Table 4 summarizes the exposure and outcome variables of the included studies. The analysis revealed several recurring themes, including the epidemiology of dental diseases, patterns of healthcare utilization, efficacy of dental treatments, and impact of oral health policies. The most frequently examined variables were dental visits or procedures, oral and maxillofacial surgery, periodontal disease, and dental caries or preventive care, which were used as exposure or outcome variables.

**Table 4:** Summary of exposure and outcome variables in included studies.

Oral health/dental practice category	Systemic health association	*n*
Dental visit/procedure	Total	107
	Cancer	2
	Cardiovascular disease	3
	Diabetes mellitus	1
	Health care cost	1
	Pregnancy	2
	Rheumatoid arthritis	1
	Others	3
Oral and maxillofacial surgery	Total	82
	Cancer	2
	Cardiovascular disease	3
	Health care cost	1
	Osteonecrosis of the jaw	10
	Osteoporosis	2
	Respiratory disease	5
	Others	6
Periodontal disease	Total	76
	Allergic rhinitis	2
	Cancer	7
	Cardiovascular disease	8
	Cerebrovascular disease	1
	Dementia	3
	Diabetes mellitus	8
	Health care cost	3
	Hypertension	1
	Kidney disease	2
	Osteonecrosis of the jaw	2
	Osteoporosis	3
	Pregnancy	3
	Respiratory disease	4
	Rheumatoid arthritis	4
	Others	20
Dental caries/preventive care	Total	64
	Allergic rhinitis	1
	Cardiovascular disease	1
	Diabetes mellitus	1
	Health care cost	1
	Osteonecrosis of the jaw	1
	Others	3
Prosthesis	Total	5
	Cardiovascular disease	1
	Others	1
Cost	Total	39
	Health care cost	2
Health policy/program	Total	40
	Cancer	1
Others	Total	68
	Cancer	2
	Rheumatoid arthritis	1
	Others	4

*Note:* Rows labeled “Total” indicate all studies in the corresponding oral/dental category. Oral/dental categories are not mutually exclusive. Therefore, a single study may include more than one oral/dental category. Rows listing systemic health associations are also nonmutually exclusive subcategories and, therefore, do not add up to the corresponding Total. Reference IDs are listed in Table S4.

Geographical trends were evident in the research focus; studies on dental visits and procedures were predominantly conducted in the United States, whereas investigations into periodontal disease and oral and maxillofacial conditions were more common in Taiwan. Research on healthcare costs was conducted primarily in the United States and Japan.

In the examination of periodontal disease and its association with systemic conditions, respiratory disease was the most extensively studied comorbidity, followed by diabetes mellitus. The range of oral health conditions examined in relation to systemic diseases demonstrates the versatility of dental claims databases.

### 3.4. Statistical Methods

The included studies used diverse statistical methods, reflecting the heterogeneity of the research questions and the complexity of data in administrative claims databases. Logistic regression was the most commonly used method to assess the association between various factors and dental health outcomes. Cox regression analysis was preferred for longitudinal investigations of time‐to‐event data, such as the duration of dental interventions.

Propensity score matching was used to balance the measured covariates between the comparison groups, thereby mitigating confounding by indication. Additionally, quasiexperimental methods were used to evaluate causal relationships and treatment effects in the absence of randomized controlled trials (RCTs).

However, designs and analytical strategies specifically developed to address unmeasured confounding factors were uncommon. Interrupted time‐series designs (*n* = 4) and simulation studies (*n* = 1) each accounted for less than 1% of the included studies. Difference‐in‐differences and self‐controlled designs were applied in nine and four studies, respectively. Studies linking claims data to at least one additional data source accounted for 254 (53.8%) of the 472 included studies (Table S2), and four studies were formal validations of claims‐based definitions. The methodological composition of the studies did not change significantly during the study period. Studies eligible for NOS appraisal—that is, cohort and case–control designs—together accounted for 47.3% of studies published in the earlier half of the period (2010–2017) and 56.5% in the latter half (2018–2025). The growth shown in Figure 2 reflects an increase in the volume of research rather than a shift in methods.

### 3.5. Risk of Bias of Included Studies

Of the 472 included studies, 253 were eligible for NOS assessment, while the remaining 219 used designs for which the instrument was not applicable and were not appraised (Figure 3). Overall, most studies were rated as low risk (83%), with no studies classified as high risk. At the domain level, most studies were at low risk in the selection domain (67%), whereas the outcome/exposure domain had the highest proportion of moderate‐risk ratings (63%). The comparability domain showed the highest variability, with 49% rated as low risk, 47% as moderate risk, and 4% as high risk. The study‐level NOS ratings are presented in Table S5.

**Figure 3:**
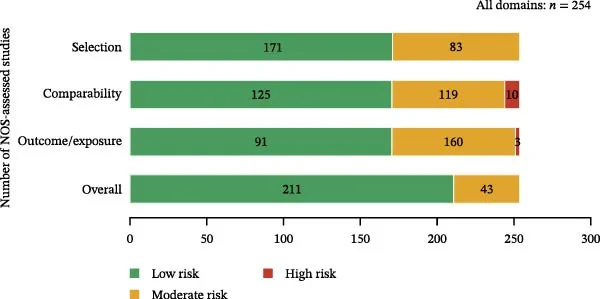
Risk of bias among studies assessed using the Newcastle–Ottawa Scale.

### 3.6. Limitations of Included Studies

The included studies reported limitations related to data quality, misclassification, residual confounding, generalizability, and limited clinical detail available in administrative claims. Periodontal pocket depth, tooth‐level findings, radiographic information, and detailed dental charts were generally unavailable. Consequently, claims‐based definitions may not accurately capture disease severity or clinical status, and associations may remain confounded by behavioral and socioeconomic factors that are not recorded in the claims data.

## 4. Discussion

This scoping review demonstrated a marked geographical concentration of dental administrative claims database research, predominantly in the United States and Taiwan, with some presence in European countries, Japan, South Korea, and Canada. This distribution reflects the robust infrastructure of healthcare data in these countries. In the medical field, advances in the use of claims databases have been reported in the United States [13], United Kingdom [14], Germany [15], France [16], Canada [17], Taiwan [18], and Japan [1, 19]. Dental research has followed a similar trend, although Canada and France have contributed less work. The United States, with the most studies included in this scoping review, benefits from multiple datasets, including Medicare, Medicaid, state‐specific data, and data from large private companies. Taiwan’s single insurance system simplifies data collection, and the availability of government‐sponsored datasets and research‐specific cohort data further supports its extensive research utilization [18]. Japan has several databases, including private and NHI data and the comprehensive NDB. With dental care covered by Japan’s universal health insurance, dental claims data are available. Although medical research in the United Kingdom thrives because of the availability of general practitioner data from the Clinical Practice Research Datalink (CPRD), there is no equivalent database for dental research. This disparity in dental and medical research applications of claims databases may mirror the historical evolution of these resources within medical research domains.

The concentration of this literature in a small number of high‐income countries may reflect structural barriers rather than differences in research interests. We argue that the secondary use of dental claims data requires at least three conditions. Dental care must fall within the insured benefit package, without which dental encounters generate no claims record; claims processing must be centralized and electronic; and an established legal and administrative route must exist by which researchers may obtain access. Every country represented in this review satisfies all three criteria. In contrast, in most low‐ and middle‐income countries, dental care is financed predominantly out of pocket and lies outside the insured benefit package, claims processing is fragmented across multiple payers or remains partly paper‐based, and frameworks governing the secondary use of administrative health data are frequently absent. Under these conditions, the absence of dental claims research reflects the absence of an underlying data infrastructure rather than the absence of research capacity or need. The geographical distribution reported here should not be interpreted as a map of where oral health research is required. Until coverage and data infrastructure develop, population‐level oral health surveillance in these settings will continue to depend on alternative sources, such as national oral health surveys and facility‐based registries.

The principal methodological opportunity in this field lies within this link. The linkage of claims data to electronic dental records, disease and procedure registries, imaging repositories, and cohort or survey data would precisely provide the clinical detail that the claims lack. A small number of included studies have demonstrated that such a linkage is feasible when identifiers and governance arrangements permit. Achieving this will require a standardized representation of dental findings, including standardized oral examination code sets and general healthcare interoperability standards, along with stable individual identifiers and legal frameworks permitting record linkage. These are infrastructural rather than analytical problems, and their progress will determine how far claims‐based dental research can move beyond describing its utilization toward studying clinical outcomes.

The number of studies included increased over the study period. Data from the Centers for Medicare and Medicaid Services in the United States have been available since 1995, from the NHI Research Database in Taiwan since 1996, and from the KDB in Japan since 1997. Increased capacity for data storage and improvements in computing power have facilitated the rapid generation and availability of large administrative claims databases [20].

The included studies covered a broad range of topics, including the epidemiology of dental diseases such as periodontal disease and dental caries, oral and maxillofacial conditions, dental treatment outcomes, patterns of dental service utilization, and the impacts of various health policies and programs on dental health. Administrative claims databases offer several advantages. They link medical and dental visits for the same individual; their large sample sizes capture rare conditions; they cover both regional and national populations, allowing policy effects to be examined; and they directly record outcomes for oral and maxillofacial conditions. These advantages have contributed to frequent investigation of these themes and reflect the multifaceted nature of dental health research [21].

The included studies varied in design, with cohort studies being the most common, followed by cross‐sectional and case–control studies. The studied populations were diverse, ranging from broadly defined groups to specific demographic subgroups. This diversity, together with the varied analytical methods and data‐linkage approaches, demonstrates the flexibility of dental claims databases in addressing a wide range of research questions.

The risk‐of‐bias assessment indicated that the majority of NOS‐eligible studies were rated as low risk overall. The selection domain showed a particularly low risk, consistent with the nature of administrative claims data, which rely on routinely collected records rather than active participant recruitment, thereby minimizing selection bias. However, demonstrating the absence of the outcome at baseline may be challenging in studies on chronic disease incidence. The outcome/exposure domain was most often rated as moderate, reflecting the difficulty of confirming outcome ascertainment and the completeness of follow‐up from claims records alone. In contrast, the comparability domain showed greater variability, reflecting the unmeasured confounding inherent to observational research.

However, these databases have limitations, including potential inaccuracy, bias, limited data fields, and threats to the validity of the findings [22, 23]. According to previous research on the Medicare database [13], databases in many countries lack data on behavioral characteristics such as nutrition and physical activity, consumption of alcohol and tobacco, laboratory data, and disease severity such as cancer staging. As many of these factors are potential confounders, comparative effectiveness analyses performed using these data may be subject to residual confounding. To address this lack of information, databases in some countries can be linked to other data sources, such as the cancer registries available in Taiwan and France and the Registry of Catastrophic Illness in Taiwan. Several attempts have been made to develop databases by expanding electronic dental record systems to capture clinical data [24].

To mitigate unmeasured confounding bias, it is essential to adopt new research designs and statistical analysis methods for causal inference. For example, self‐controlled designs, such as self‐controlled case series and case‐crossover analyses, control for time‐invariant unmeasured confounding within individuals. In this review, only a small number of the included studies employed a self‐controlled design. Quasiexperimental designs, such as the difference‐in‐differences approach [25, 26], address such confounding through a comparison group with a parallel outcome trend. Despite the development of these new statistical methods, a substantial portion of the literature still relies on traditional techniques, such as logistic and Cox regression analyses, which are potentially vulnerable to unmeasured confounding bias. A Cochrane review reported little to no difference in effect estimates between RCTs and observational studies; however, the differences were significant when adjustment for confounding was insufficient [27]. Appropriate statistical methods that effectively address confounding should be promoted in dentistry.

Regarding the limitations of data validity, many validation studies on disease and procedure codes in claims data have been conducted in several countries [28, 29]. However, most have focused on specific medical disease or procedure codes, with few examining the validity of specific dental claims data. A study in Japan assessed the validity of dental procedure codes, the number of teeth recorded in dental claims, and dental disease codes [30]. Since the assignment of disease and procedure codes can vary by country, conducting additional validity studies in countries using dental administrative data for research would strengthen the data. Furthermore, to improve disease and outcome definitions and avoid unnecessary replication of previous work, repositories for the codes used in past clinical research are being developed in some countries, including the United Kingdom [31] and Japan [32].

This study had several limitations. First, the search used generic terms for claims and administrative data rather than an enumerated list of proprietary database names; studies that identified their data source only by proprietary names, without generic descriptors in the title, abstract, or indexing terms, may not have been retrieved. Second, agreement between reviewers at the screening stage was not quantified, and reviewer‐level decisions were not retained in a form permitting retrospective calculation. Third, the clinical topic and systemic condition categories were developed inductively during charting rather than prespecified. Thus, the boundaries of the “other” category in particular reflect the composition of the retrieved literature rather than an external taxonomy. Fourth, whether a study validated its claims‐based definitions was not a prespecified charting item. Therefore, the small number of validation studies identified here should be read as a lower bound. Fifth, eligibility was restricted to articles published in English or Japanese, which might have accentuated the aforementioned geographical concentration.

In conclusion, this scoping review demonstrates the expanding yet geographically concentrated use of dental claims databases across 16 countries. Although the quality of the studies was generally high, common limitations included concerns about the validity of diagnoses and procedures and the lack of detailed clinical information. Future research should prioritize validating claims‐based diagnosis and procedure codes and linking claims data with clinical records. A greater use of longitudinal data and causal inference methods is required to fully leverage the potential of dental claims databases to advance oral health research and improve healthcare outcomes.

## Author Contributions

Misuzu Sato and Miho Ishimaru contributed to the conception, design, data acquisition, and interpretation of the study and drafted the manuscript. Sachiko Ono and Yusuke Ida contributed to the conception, design, data acquisition, and interpretation of the study and critically revised the manuscript.

## Funding

This work was supported by grants from the Ministry of Health, Labour and Welfare, Japan (Grants 23FA1022 and 26FA1014) and the Ministry of Education, Culture, Sports, Science and Technology, Japan (Grants 24K20093, 23K19735, and 22K17284).

## Disclosure

After ChatGPT, the authors reviewed and edited the content as needed and take full responsibility for the content of the publication. All authors gave their final approval and agree to be accountable for all aspects of the work.

## Conflicts of Interest

The authors declare no conflicts of interest.

## Supporting Information

Additional supporting information can be found online in the Supporting Information section.

## Supporting information


**Supporting Information** Table S1: All extracted studies by country. Table S2: Standardized database groups, original database names, oldest research year, linked databases, and references. Table S3: Study designs of included studies with reference IDs. Table S4: Grouped exposure and outcome variables in included studies with reference IDs. Table S5: Study‐level Newcastle–Ottawa Scale risk‐of‐bias ratings. Appendix 1: Standardized prompt used to generate preliminary Newcastle–Ottawa Scale (NOS) ratings.

## Data Availability

This scoping review is based solely on the published literature and does not involve any primary data analysis. All studies included in this review are available in the public domain and can be accessed through their respective publishers or databases, as cited in the reference list.
