# Supplementary material for: Administrative Claims Databases in Dentistry: Current Applications, Methodological Trends, and Evidence Gaps: A Global Scoping Review of Dataset Characteristics
Source: Int J Dent. 2026 Oct 19;2026:8870016. doi: 10.1155/ijod/8870016 (PMC13680800; doi:10.1155/ijod/8870016)
Supplement: Supplementary file 1 [file IJOD-2026-8870016-s001.docx]

**Supplementary files of**

**“Administrative Claims Databases in Dentistry: Current Applications, Methodological Trends, and Evidence Gaps—A Global Scoping Review of Dataset Characteristic”**

**Supplementary Material legends**

**Supplementary Table 1.** All extracted studies by country.

**Supplementary Table 2.** Standardized database groups, original database names, oldest research year, linked databases, and references.

**Supplementary Table 3.** Study designs of included studies with reference IDs.

**Supplementary Table 4.** Grouped exposure and outcome variables in included studies with reference IDs.

**Supplementary Table 5.** Study-level Newcastle-Ottawa Scale risk-of-bias ratings.

**Supplementary Appendix 1.** Standardized prompt used to generate preliminary Newcastle-Ottawa Scale (NOS) ratings.

**Supplementary Table 1. All extracted studies by country.**

| United States | | | |
| --- | --- | --- | --- |
| No | year | title | First Author |
| US-1 | 2010 | Invasive dental treatment and risk for vascular events: a self-controlled case series. | Minassian C |
| US-2 | 2010 | Cross-sectional study of periodontal care and Glycosylated Hemoglobin in an insured population. | Spangler L |
| US-3 | 2010 | Doctor, my tooth hurts: the costs of incomplete dental care in the emergency room. | Davis EE |
| US-4 | 2010 | Geographic variation in medicaid claims for dental procedures in New York State: role of fluoridation under contemporary conditions. | Kumar JV |
| US-5 | 2011 | Dental procedures and subsequent prosthetic joint infections: findings from the Medicare Current Beneficiary Survey. | Skaar DD |
| US-6 | 2011 | Impact of chronic condition status and severity on dental utilization for Iowa Medicaid-enrolled children. | Chi DL |
| US-7 | 2011 | Dental utilization by adult Medicaid enrollees who have indicators of intellectual and developmental disabilities (IDD). | Chalmers JM |
| US-8 | 2011 | The individual and program impacts of eliminating Medicaid dental benefits in the Oregon Health Plan. | Wallace NT |
| US-9 | 2011 | The influence of comorbidity and other health measures on dental and medical care use among Medicare beneficiaries 2002. | Chen H |
| US-10 | 2011 | Patient and plan characteristics affecting abandonment of oral oncolytic prescriptions. | Streeter SB |
| US-12 | 2011 | An examination of periodontal treatment, dental care, and pregnancy outcomes in an insured population in the United States. | Albert DA |
| US-13 | 2011 | The Effects of Medicaid and CHIP Policy Changes on Receipt of Preventive Care among Children | Kenney |
| US-14 | 2012 | Dental procedures and risk of experiencing a second vascular event in a Medicare population. | Skaar D |
| US-16 | 2012 | Cost-effectiveness of preventive oral health care in medical offices for young Medicaid enrollees. | Stearns SC |
| US-17 | 2012 | The cost burden of oral, oral pharyngeal, and salivary gland cancers in three groups: commercial insurance, Medicare, and Medicaid. | Jacobson JJ |
| US-18 | 2012 | Rural-urban differences in dental service use among children enrolled in a private dental insurance plan in Wisconsin: analysis of administrative data. | Bhagavatula P |
| US-19 | 2012 | Evaluating components of dental care utilization among adults with diabetes and matched controls via hurdle models. | Chaudhari M |
| US-20 | 2012 | Rural-urban differences in dental service utilization among an early childhood population enrolled in South Carolina Medicaid. | Martin AB |
| US-21 | 2012 | Hospitalizations primarily attributed to dental conditions in the United States in 2008 | Allareddy |
| US-22 | 2012 | Factors associated with length of stay and hospital charges for patients hospitalized with mouth cellulitis | Kim MK |
| US-23 | 2012 | Burden of facial cellulitis: estimates from the Nationwide Emergency Department Sample | Kim MK |
| US-24 | 2013 | Geographic accessibility and utilization of orthodontic services among Medicaid children and adolescents. | McKernan SC |
| US-25 | 2013 | Threshold analysis of reimbursing physicians for the application of fluoride varnish in young children. | Hendrix KS |
| US-26 | 2013 | Factors affecting age at first dental exam for Medicaid-enrolled children seen at Federally Qualified Health Centers. | Kuthy RA |
| US-27 | 2013 | Periodicity of dental recall visits for young children first seen in community health centers. | Kuthy RA |
| US-28 | 2013 | Evaluating the impact of patient, tumor, and treatment characteristics on the development of jaw complications in patients treated for oral cancers: a SEER-Medicare analysis. | Beadle BM |
| US-29 | 2013 | Regional epidemiologic assessment of prevalent periodontitis using an electronic health record system. | Acharya A |
| US-30 | 2013 | The impact of a home visiting program on children's utilization of dental services. | Brickhouse TH |
| US-31 | 2013 | Children's utilization of the U.S. military dental insurance. | Chaffin JG |
| US-32 | 2013 | A Serial Cross-sectional Study of Pediatric Inpatient Hospitalizations for Non-traumatic Dental Conditions | Chi DL |
| US-33 | 2013 | The characteristics of hospital emergency department visits made by people with mental health conditions who had dental problems | Nalliah RP |
| US-34 | 2013 | Accuracy of record linkage software in merging dental administrative data sets | Beil H |
| US-35 | 2014 | Age and stage as determinants of treatment for oral cavity and oropharyngeal cancers in the elderly. | Goldenberg D |
| US-36 | 2014 | Impact of periodontal therapy on general health: evidence from insurance data for five systemic conditions. | Jeffcoat MK |
| US-37 | 2014 | North Carolina physician-based preventive oral health services improve access and use among young Medicaid enrollees. | Kranz AM |
| US-39 | 2014 | Cost-effectiveness of pit-and-fissure sealants on primary molars in Medicaid-enrolled children. | Chi DL |
| US-40 | 2014 | Time until first dental caries for young children first seen in Federally Qualified Health Centers: a retrospective cohort study. | Kuthy RA |
| US-41 | 2014 | Cost analysis of dental services needed before hematopoietic cell transplantation | Mawardi |
| US-42 | 2014 | Racial/ethnic disparities in provision of dental procedures to children enrolled in Delta Dental insurance in Milwaukee, Wisconsin | Bhagavatula |
| US-43 | 2015 | The Validity of Claims-Based Algorithms to Identify Serious Hypersensitivity Reactions and Osteonecrosis of the Jaw. | Wright NC |
| US-44 | 2015 | Accuracy of administrative and clinical registry data in reporting postoperative complications after surgery for oral cavity squamous cell carcinoma. | Awad MI |
| US-45 | 2015 | Impact of an interprofessional oral health education program on health care professional and practice behaviors: a RE-AIM analysis. | Braun PA |
| US-46 | 2015 | Measuring quality of dental care: Caries prevention services for children. | Herndon JB |
| US-47 | 2015 | Emergency department visits for nontraumatic dental problems: a mixed-methods study. | Sun BC |
| US-48 | 2015 | Geographic variation of dental utilization among low income children. | McKernan SC |
| US-49 | 2015 | The effect of Medicaid primary care provider reimbursement on access to early childhood caries preventive services. | Herndon JB |
| US-50 | 2015 | Aspiration Pneumonia After Concurrent Chemoradiotherapy for Head and Neck Cancer | Xu B |
| US-51 | 2016 | Preventive Dental Care and Long-Term Dental Outcomes among ALL Kids Enrollees. | Sen B |
| US-52 | 2016 | Dental care after an emergency department visit for dental problems among adults enrolled in Medicaid. | Singhal A |
| US-53 | 2016 | Dental utilization for Medicaid-enrolled children with cystic fibrosis. | Sarvas EW |
| US-54 | 2016 | Potentially preventable dental care in operating rooms for children enrolled in Medicaid. | Bruen BK |
| US-55 | 2016 | Impact of a Community Dental Access Program on Emergency Dental Admissions in Rural Maryland. | Rowl S |
| US-56 | 2016 | Adjuvant chemoradiotherapy versus with radiotherapy alone for locally advanced salivary gland carcinoma among older patients. | Tanvetyanon T |
| US-57 | 2016 | Travel burden and dentist bypass among dentally insured children. | McKernan SC |
| US-58 | 2016 | Efficacy of Elective Neck Dissection in T1/T2N0M0 Oral Tongue Squamous Cell Carcinoma: A Population-Based Analysis. | Patel TD |
| US-59 | 2016 | Travel distances by Wisconsin Medicaid enrollees who visit emergency departments for dental care. | Okunseri C |
| US-60 | 2016 | Dental Visits by Age One: General Dentist Availability for Privately Insured Children in a Rural State. | McKernan SC |
| US-61 | 2016 | Lymph Node Yield as a Predictor of Survival in Pathologically Node Negative Oral Cavity Carcinoma. | Lemieux A |
| US-62 | 2016 | Impact of South Carolina's Medicaid fluoride varnish reimbursement policy on children's receipt of fluoride varnish in medical and dental settings | Veschusio |
| US-63 | 2017 | Association Between Employee Dental Claims, Health Risks, Workplace Productivity, and Preventive Services Compliance. | Burton WN |
| US-64 | 2017 | National Database Analysis of Single-Level versus Multilevel Sleep Surgery. | Brietzke SE |
| US-65 | 2017 | Outcomes Associated With Early Preventive Dental Care Among Medicaid-Enrolled Children in Alabama. | Blackburn J |
| US-66 | 2017 | Measuring quality: caries-related emergency department visits and follow-up among children. | Herndon JB |
| US-67 | 2017 | Differences in utilization of dental procedures by children enrolled in Wisconsin Medicaid and Delta Dental insurance plans. | Bhagavatula P |
| US-69 | 2017 | The Relationship between Periodontal Interventions and Healthcare Costs and Utilization. Evidence from an Integrated Dental, Medical, and Pharmacy Commercial Claims Database. | Nasseh K |
| US-70 | 2017 | Using the Beers criteria to identify potentially inappropriate medication use by older adult dental patients. | Skaar DD |
| US-71 | 2017 | "They Told Me to Take Him Somewhere Else": Caregivers' Experiences Seeking Emergency Dental Care for Their Children. | Meyer BD |
| US-72 | 2017 | An evaluation of dental antibiotic prescribing practices in the United States | Durkin |
| US-73 | 2018 | Oral Health Needs and Experiences of Medicaid Enrollees With Serious Mental Illness. | Hall JP |
| US-75 | 2018 | Trends and Characteristics of Pediatric Dentistry Patients Treated under General Anesthesia. | Rudie MN |
| US-76 | 2018 | Implementing an early childhood oral health program in a federally qualified health center in North Carolina. | Quinonez RB |
| US-77 | 2018 | Estimating the Cost Savings of Preventive Dental Services Delivered to Medicaid-Enrolled Children in Six Southeastern States. | Lee I |
| US-78 | 2018 | Evidence of effectiveness of preventive dental care in reducing dental treatment use and related expenditures. | Pourat N |
| US-79 | 2018 | The Effects of Medical Well Baby Visits in Promoting Earlier First Dental Visits for Children. | Park S |
| US-80 | 2018 | History of periodontal treatment and risk for intrauterine growth restriction (IUGR). | Ananth CV |
| US-81 | 2018 | Persistent Opioid Use After Wisdom Tooth Extraction. | Harbaugh CM |
| US-83 | 2018 | Spillovers from the Patient Protection and Affordable Care Act to employer-sponsored dental insurance enrollment | Huang S |
| US-84 | 2018 | Survival of patients with head and neck cancer treated with definitive radiotherapy and concurrent cisplatin or concurrent cetuximab: A Surveillance, Epidemiology, and End Results-Medicare analysis | Xiang M |
| US-85 | 2018 | Assessment of inappropriate antibiotic prescribing among a large cohort of general dentists in the United States | Durkin MJ |
| US-86 | 2018 | Dental safety net capacity: An innovative use of existing data to measure dentists' clinical engagement in state Medicaid programs | Maxey HL |
| US-88 | 2018 | Opioid prescribing practices from 2010 through 2015 among dentists in the United States What do claims data tell us? | Gupta N |
| US-89 | 2019 | Assessment of the Appropriateness of Antibiotic Prescriptions for Infection Prophylaxis Before Dental Procedures, 2011 to 2015. | Suda KJ |
| US-90 | 2019 | Billed and Paid Amounts for Preventive Procedures in Dental Medicaid. | Wagner K |
| US-91 | 2019 | Delivery of Preventive Oral Health Services by Rurality: A Cross-Sectional Analysis. | Geiger CK |
| US-92 | 2019 | Short- and Long-term Opioid Use in Patients with Oral and Oropharynx Cancer. | McDermott JD |
| US-94 | 2019 | Emergency department revisits for nontraumatic dental conditions in Massachusetts. | Ranade A |
| US-95 | 2019 | Utilization of Stainless Steel Crowns by Pediatric and General Dentists. | Shelton A |
| US-96 | 2019 | Association between Medical Well-Child Visits and Dental Preventive Visits: A Big Data Report. | Tiwari T |
| US-97 | 2019 | Feasibility and usability of measuring receipt of sealants in 2 states. | Shenkman E |
| US-98 | 2019 | Rates of sialoendoscopy and sialoadenectomy in 5,111 adults with private insurance. | Vila PM |
| US-99 | 2019 | Trends in Medicaid-funded adolescent comprehensive orthodontic treatment provided by orthodontists to children in Oklahoma between 2010 and 2016. | Lamb C |
| US-102 | 2019 | Filled dietary fluoride supplement prescriptions for Medicaid-enrolled children living in states with high and low water fluoridation coverage. | Griffin SO |
| US-104 | 2019 | Prevalence of Comorbidities among Older Head and Neck Cancer Survivors in the United States | Eytan DF |
| US-109 | 2020 | Emergency and urgent dental visits among Medicaid enrollees from 2013 to 2017. | Fiehn R |
| US-110 | 2020 | Dental sealants and restorative treatment for first molars among Medicaid enrollees. | Okunseri CE |
| US-111 | 2020 | Changes in Dental Benefits and Use of Emergency Departments for Nontraumatic Dental Conditions in Massachusetts. | Ranade A |
| US-112 | 2020 | The effect of an accountable care organization on dental care for children with disabilities. | Song PH |
| US-113 | 2020 | Dental visits in Medicaid-enrolled youth with mental illness: an analysis of administrative claims data. | Stockbridge EL |
| US-114 | 2020 | Overprescribing of Opioids to Adults by Dentists in the U.S., 2011-2015. | Suda KJ |
| US-116 | 2020 | Dental Services Utilization by Rurality Among Privately Insured Children in the United States. | Simon L |
| US-118 | 2020 | A Cross-Sectional Analysis of Community Water Fluoridation and Prevalence of Pediatric Dental Surgery Among Medicaid Enrollees. | Lee HH |
| US-120 | 2020 | The Incidence of Root Canal Therapy after Full-Coverage Restorations: A 10-Year Retrospective Study. | Yavorek A |
| US-123 | 2020 | Demonstration of payer readiness for value-based care in a fee-for-service environment: Measuring provider performance on sealant delivery. | Martin A |
| US-124 | 2020 | Complications and the Need for Long-Term Follow-Up after Secondary Speech Surgery: A National and Longitudinal Claims Analysis. | Makar KG |
| US-126 | 2020 | Success Rates of Pulpotomies Performed by General Dentists Versus Pediatric Dentists: A Claims Data Analysis. | Yepes JF |
| US-127 | 2020 | A Machine Learning Approach to Uncovering Hidden Utilization Patterns of Early Childhood Dental Care Among Medicaid-Insured Children. | Peng J |
| US-129 | 2021 | Dental services use before and after inpatient admission among privately insured adults in the United States. | Simon L |
| US-130 | 2021 | Age of First Oral Health Examination and Dental Treatment Needs of Medicaid-Enrolled Children. | Ahmed I |
| US-131 | 2021 | Variation in dental services by rurality among privately insured adults in the United States. | Simon L |
| US-132 | 2021 | Association between maternal periodontal disease and adverse pregnancy outcomes: an analysis of claims data. | Choi SE |
| US-133 | 2021 | Emergency department visits for dental problems among adults with private dental insurance: A national observational study. | Amen TB |
| US-135 | 2021 | Racial/Ethnic Differences in Receipt of Oral Health Services in Medical and Dental Offices: Impact of Medicaid Policies on Young Children. | Kranz AM |
| US-136 | 2021 | Benefits of the Involvement of Dentists in Managing Oral Complications Among Patients With Oral Cavity and Oropharyngeal Cancer: An Analysis of Claims Data. | Choi SE |
| US-137 | 2021 | Preventive oral health care use for children with special health care needs aged 6 through 12 years enrolled in Medicaid: A mixed methods study. | Lang C |
| US-138 | 2021 | The Effect of Well Child Visit Location on Preventative Dental Visit. | Tiwari T |
| US-140 | 2021 | Dental care utilization in Massachusetts before and after initiation of medication for opioid use disorder: A cross-sectional study of a state all-payer claims database. | Simon L |
| US-141 | 2021 | Association between first oral examination characteristics and dental treatment needs in privately insured children: A claims data analysis. | McGivern S |
| US-142 | 2021 | Impact of state-level training requirements for medical providers on receipt of preventive oral health services for young children enrolled in Medicaid. | Goldstein EV |
| US-145 | 2022 | Oral Health and Academic Achievement of Children in Low-Income Families. | Wehby GL |
| US-146 | 2022 | Periodontal Disease and Risk of Dementia in Medicare Patients with Hepatitis C Virus. | Malone J |
| US-147 | 2022 | Does preventive dental care reduce nonpreventive dental visits and expenditures among Medicaid-enrolled adults? | Taylor HL |
| US-148 | 2022 | Variation in prices for an evidence-based pediatric preventive service. | Kranz AM |
| US-149 | 2022 | Beneficiaries' perspectives on improved oral health and its mediators after Medicaid expansion in Michigan: a mixed methods study. | Kieffer EC |
| US-150 | 2022 | Comparative analysis of dental procedure mix in public and private dental benefits programs. | Nasseh K |
| US-151 | 2022 | Medicaid Payment and Fluoride Varnish Application During Pediatric Medical Visits. | Kranz AM |
| US-152 | 2022 | The impact of underutilization of preventive dental care by adult Medicaid participants. | Okunev I |
| US-153 | 2022 | Cognitive impairment and edentulism among older adults: an observational study using claims data. | Choi SE |
| US-154 | 2022 | A cross-sectional analysis of oral health care spending over the life span in commercial- and Medicaid-insured populations. | Tranby EP |
| US-155 | 2022 | Prescription of bite-wing and panoramic radiographs in pediatric dental patients: An assessment of current trends and provider compliance. | Menaker NH |
| US-156 | 2022 | Initial opioid prescribing and subsequent opioid use after dental procedures among opioid-naive patients in Pennsylvania Medicaid, 2012 through 2017. | Khouja T |
| US-157 | 2022 | Measuring the quality of dental care among privately insured children in the United States | Choi SE |
| US-158 | 2021 | Determinants of Tooth Loss in a Medicaid Adult Population | Tiwari T |
| US-160 | 2024 | Multilevel Interventions and Dental Attendance in Pediatric Primary Care A Cluster Randomized Clinical Trial | Nelson S |
| US-161 | 2024 | Association between dental visits during pregnancy and setting for prenatal care | Byrappagari D |
| US-162 | 2023 | Healthcare Utilization Among Children Receiving Permanent Supportive Housing | Bohnhoff JC |
| US-163 | 2022 | Racial and ethnic disparities in oral healthcare quality among children enrolled in Medicaid and CHIP | Herndon JB |
| US-169 | 2025 | The Effect of a Rural Workforce Intervention to Improve Oral Health of Children: Evidence From an Interrupted Time Series Study of Southeast Alaska. | Pourat N |
| US-170 | 2025 | Changes in Children's Receipt of Fluoride Varnish During Medical Visits and the COVID-19 Pandemic. | Kranz AM |
| US-171 | 2025 | Racial Disparities in Quality of Dental Care Among Publicly Insured Children. | Choi SE |
| US-172 | 2025 | Risk of Oral Health Problems in Adults With Opioid Use Disorder Treated With Transmucosal Buprenorphine. | Tuan WJ |
| US-173 | 2025 | Pandemic-driven shift: increase in silver diamine fluoride utilization among Medicaid-enrolled children during the COVID-19 public health emergency. | Meyer BD |
| US-174 | 2023 | Association Between Insurance Type and Fluoride Varnish Application During Well-Child Visits in Massachusetts. | Chen AYA |
| US-177 | 2022 | Serious opioid-related adverse outcomes associated with opioids prescribed by dentists. | Khouja T |
| US-180 | 2025 | Medicaid Payments and Outcomes for Pediatric Dental Surgical Procedures by Site of Care. | Kranz AM |
| US-181 | 2025 | The Association of Medical Periodicity Adherence and Early Preventive Dental Care With Subsequent Dental Utilization Among Medicaid-Insured Ohio Children. | Higgins A |
| US-182 | 2025 | Predictive Modeling of Sjögren's Disease Using US Healthcare Claims Data. | Mao J |
| US-184 | 2025 | Oral cavity and oropharyngeal cancers in Texas: examining incidence rates in dental health professional shortage areas. | Griner SB |
| US-185 | 2024 | Affordable Care Act's Preventive Services Coverage Mandate and Receipt of Fluoride Varnish. | Kranz AM |
| US-187 | 2025 | Predicting Dental General Anesthesia Use among Children with Behavioral Health Conditions. | Peng J |
| US-189 | 2024 | Longitudinal Follow-up Comparing Future Care of Primary Teeth Treated with Silver Diammine Fluoride: A Private Dental Claims Review. | Schlotz JC |
| US-190 | 2024 | Age of first dental visits: A benefit of the pediatric medical home. | Bussard N |
| US-191 | 2024 | Barriers to dental utilization among Medicaid-enrolled young children from primary care practices in Northeast Ohio. | Selvaraj D |
| US-192 | 2024 | Eliminating Medicaid dental benefits and early-stage oral cancer diagnoses. | Semprini J |
| US-193 | 2024 | Molecular Profiling and the Impact of Treatment on Outcomes in Adenoid Cystic Carcinoma Type I and II. | Hanna GJ |
| US-194 | 2024 | The relationship between preventive dental care and overall medical expenditures. | Taylor HL |
| US-195 | 2024 | Measuring quality of dental care during pregnancy. | Herndon JB |
| US-196 | 2024 | Inflammatory Tongue Conditions and Risk of Oral Tongue Cancer Among the US Elderly Individuals. | Tota JE |
| US-197 | 2023 | Dental Utilization of Refugee Children in Washington State Varies by Demographic Characteristics. | Seminario AL |
| US-199 | 2023 | Periodontal treatment and subsequent clinical outcomes and medical care costs: A retrospective cohort study. | Michalowicz BS |
| US-200 | 2023 | Apexification Outcomes in the United States: A Retrospective Cohort Study. | Burns LE |
| US-201 | 2023 | Amalgam or composite in pediatric dentistry: Analysis of private insurance claims data. | Dentino FC |
| US-204 | 2023 | Testing dental quality measures: Emergency department visits for nontraumatic dental conditions and subsequent follow-up dental visits. | Obadan-Udoh E |
| US-205 | 2023 | Impact of the COVID-19 pandemic on pediatric Medicaid dental claims in Arizona. | Mommaerts K |
| US-206 | 2023 | Periodontal treatment associated with decreased diabetes mellitus-related treatment costs: An analysis of dental and medical claims data. | Thakkar-Samtani M |
| US-207 | 2023 | Pediatric oral health services in Medicaid managed care and fee for service. | Chen AY |
| US-208 | 2022 | Insurance claim data trends in pulpal therapy for pediatric patients. | White LM |
| US-209 | 2023 | The Importance of Oral History: Does Dental Implant Placement or Caries One Year Before or After Primary Total Knee Arthroplasty Increase Medical Complications and Periprosthetic Joint Infections? | Gordon AM |
| US-210 | 2023 | Estimation of oral disease burden from claims and self-reported data. | Okunseri C |
| US-211 | 2023 | Dental Care Differences Among the Behavioral Health Population in an Accountable Care Organization: A Retrospective Cohort. | Peng J |
| US-212 | 2022 | Oral Cancer Prevalence, Mortality, and Costs in Medicaid and Commercial Insurance Claims Data. | Tranby EP |
| US-213 | 2022 | Characteristics of Medical Malpractice Claims Involving Temporomandibular Joint Surgery in the United States. | Green MA |
| US-214 | 2023 | The Impact of Preventive Dental Services on Subsequent Dental Treatment for Children in Medicaid. | Brickhouse TH |
| US-223 | 2025 | Examination of dental utilization of newly resettled adult refugees in Washington state enrolled in dental medicaid program | Seminario AL |
| US-226 | 2025 | Pandemic-Associated Dental Office Closures Associated With Increased Use of Emergency Departments for Dental Conditions in Publicly Insured Children In New York State | Huang SS |
| US-228 | 2022 | Appropriateness of Antibiotic Prophylaxis Before Dental Procedures, 2016-2018. | Hubbard CC |

| Taiwan | | | |
| --- | --- | --- | --- |
| TW-1 | 2011 | Incidence and mortality of treated primary Sjogren's syndrome in Taiwan: a population-based study. | Weng MY |
| TW-2 | 2011 | The risk of temporomandibular disorder in patients with depression: a population-based cohort study. | Liao CH |
| TW-3 | 2012 | Positive association between hepatitis C infection and oral cavity cancer: a nationwide population-based cohort study in Taiwan. | Su FH |
| TW-4 | 2013 | Progression of precancerous lesions to oral cancer: results based on the Taiwan National Health Insurance Database. | Lian IeB |
| TW-5 | 2013 | Sudden sensorineural hearing loss associated with chronic periodontitis: a population-based study. | Wu CS |
| TW-6 | 2013 | Association between ankylosing spondylitis and chronic periodontitis: a population-based study. | Keller JJ |
| TW-7 | 2013 | Association between a history of periodontitis and the risk of rheumatoid arthritis: a nationwide, population-based, case-control study. | Chen HH |
| TW-9 | 2013 | Periodontitis and etanercept discontinuation risk in anti-tumor necrosis factor-naive rheumatoid arthritis patients: a nationwide population-based cohort study. | Chen HH |
| TW-10 | 2014 | Pioglitazone and oral cancer risk in patients with type 2 diabetes. | Tseng CH |
| TW-11 | 2014 | The national-scale cohort study on bisphosphonate-related osteonecrosis of the jaw in Taiwan. | Yuh DY |
| TW-12 | 2014 | Cancer risk among gingivitis and periodontitis patients: a nationwide cohort study. | Wen BW |
| TW-13 | 2014 | Increased dental visits in patients prior to diagnosis of primary Sjogren's syndrome: a population-based study in Taiwan. | Lu MC |
| TW-14 | 2014 | Non-apnea sleep disorder increases the risk of periodontal disease: a retrospective population-based cohort study. | Lee CF |
| TW-15 | 2014 | Periodontitis exposure within one year before anti-diabetic treatment and the risk of rheumatoid arthritis in diabetes mellitus patients: a population-based cohort study. | Chen HH |
| TW-16 | 2014 | Surgical Treatment for Patients With Periodontal Disease Reduces Risk of End-Stage Renal Disease: A Nationwide Population-Based Retrospective Cohort Study | Lee CF |
| TW-17 | 2014 | Population-Based 5-Year Follow-Up Study in Taiwan of Osteoporosis and Risk of Periodontitis | Chang WP |
| TW-18 | 2015 | Association between dental amalgam fillings and Alzheimer's disease: a population-based cross-sectional study in Taiwan. | Sun YH |
| TW-19 | 2015 | Dental Procedures and the Risk of Infective Endocarditis. | Chen PC |
| TW-20 | 2015 | Dental prophylaxis decreases the risk of acute myocardial infarction: a nationwide population-based study in Taiwan. | Lee YL |
| TW-21 | 2015 | Determinants for aggressive end-of-life care for oral cancer patients: a population-based study in an Asian country. | Chang TS |
| TW-22 | 2015 | (131)I treatment for thyroid cancer and the risk of developing salivary and lacrimal gland dysfunction and a second primary malignancy: a nationwide population-based cohort study. | Ko KY |
| TW-23 | 2015 | Risk of Periodontal Diseases in Patients With Chronic Obstructive Pulmonary Disease: A Nationwide Population-based Cohort Study. | Shen TC |
| TW-24 | 2015 | Beneficial impact of multidisciplinary team management on the survival in different stages of oral cavity cancer patients: results of a nationwide cohort study in Taiwan. | Tsai WC |
| TW-25 | 2015 | Determinants of End-of-Life Expenditures in Patients with Oral Cancer in Taiwan: A Population-Based Study | Lee CC |
| TW-26 | 2016 | Sialolithiasis is associated with nephrolithiasis: a case-control study. | Wu CC |
| TW-27 | 2016 | Dental service use among patients with specific disabilities: A nationwide population-based study. | Lin PY |
| TW-28 | 2016 | Dysthymia increases the risk of temporomandibular disorder: A population-based cohort study (A STROBE-Compliant Article). | Lin SL |
| TW-29 | 2016 | Utilization of tooth filling services by people with disabilities in Taiwan. | Chen MC |
| TW-30 | 2016 | Association of sialolithiasis with cholelithiasis: A population-based study. | Hung SH |
| TW-31 | 2016 | Comparison of Elixhauser and Charlson Methods for Predicting Oral Cancer Survival. | Chang HJ |
| TW-32 | 2016 | Risk of Allergic Rhinitis, Allergic Conjunctivitis, and Eczema in Children Born to Mothers with Gum Inflammation during Pregnancy. | Hsieh VC |
| TW-33 | 2016 | Allergic Rhinitis Is Associated With Periodontitis: A Population-Based Study. | Hung SH |
| TW-34 | 2016 | Utilization of dental care among patients with severe mental illness: a study of a National Health Insurance database. | Teng PR |
| TW-35 | 2016 | The Impact of Oral Hygiene Maintenance on the Association Between Periodontitis and Osteoporosis: A Nationwide Population-Based Cross Sectional Study. | Huang YF |
| TW-36 | 2016 | Periodontal Treatment Reduces Risk of Adverse Respiratory Events in Patients With Chronic Obstructive Pulmonary Disease: A Propensity-Matched Cohort Study. | Shen TC |
| TW-37 | 2017 | Risk of Periodontal Disease in Patients With Asthma: A Nationwide Population-Based Retrospective Cohort Study. | Shen TC |
| TW-38 | 2017 | Dental Prophylaxis and Osteoradionecrosis: A Population-Based Study. | Chang CT |
| TW-39 | 2017 | Patients with chronic periodontitis present increased risk for osteoporosis: A population-based cohort study in Taiwan. | Mau LP |
| TW-40 | 2017 | Association between periodontitis and the risk of palindromic rheumatism: A nationwide, population-based, case-control study. | Lin CH |
| TW-41 | 2017 | Association between a history of periodontitis and the risk of systemic lupus erythematosus in Taiwan: A nationwide, population-based, case-control study. | Wu YD |
| TW-42 | 2017 | Temporal relationship between dysthymia and temporomandibular disorder: a population-based matched case-control study in Taiwan. | Lin SL |
| TW-43 | 2017 | Impact of periodontal treatment on hospitalization for adverse respiratory events in asthmatic adults: A propensity-matched cohort study. | Shen TC |
| TW-44 | 2017 | Influence of time interval from diagnosis to treatment on survival for oral cavity cancer: A nationwide cohort study. | Tsai WC |
| TW-45 | 2018 | Allergic rhinitis, rather than asthma, is a risk factor for dental caries. | Chuang CY |
| TW-46 | 2018 | Risk of Infective Endocarditis After Invasive Dental Treatments: Case-Only Study. | Chen TT |
| TW-47 | 2018 | Association between Peptic Ulcer Disease and Periodontitis: A Nationwide Population-Based Case-Control Study in Taiwan. | Yu HC |
| TW-48 | 2018 | Increased risk of periodontitis among patients with Crohn's disease: a population-based matched-cohort study. | Chi YC |
| TW-49 | 2018 | Patients with chronic periodontitis present increased risk for primary Sjogren syndrome: a nationwide population-based cohort study | Lin TC |
| TW-50 | 2018 | A sialoadenectomy is associated with an increased risk of coronary heart disease: A three-year follow-up study | Hung SH |
| TW-51 | 2018 | Malignant transformation of Taiwanese patients with oral leukoplakia: A nationwide population-based retrospective cohort study | Wang TY |
| TW-52 | 2019 | A case-control study of the association between sialolithiasis and osteoporosis. | Hung SH |
| TW-53 | 2019 | The association with xerostomia from sialadenitis and the jaw osteonecrosis in head and neck cancer population: a nationwide cohort study. | Huang YF |
| TW-54 | 2019 | Increased risk of tuberculosis in oral cancer patients in an endemic area: a nationwide population-based study. | Teng CJ |
| TW-55 | 2019 | Sialadenitis May Be Associated With an Increased Risk for Osteoradionecrosis: A Nationwide Population-Based Cohort Study. | Huang YF |
| TW-56 | 2019 | Increased Risk of Polycystic Ovary Syndrome in Taiwanese Women with Chronic Periodontitis: A Nationwide Population-Based Retrospective Cohort Study. | Tong C |
| TW-57 | 2019 | Trends in regional cancer mortality in Taiwan 1992-2014 | Ho YR |
| TW-58 | 2019 | Fungal Diseases in Taiwan-National Insurance Data and Estimation | Huang YR |
| TW-59 | 2020 | Examining the association between oral health status and dementia: A nationwide nested case-controlled study. | Lin JW |
| TW-60 | 2020 | Association between periodontitis and bipolar disorder: A nationwide cohort study. | Chang KH |
| TW-61 | 2020 | The association between dental therapy timelines and osteoradionecrosis: a nationwide population-based cohort study. | Huang YF |
| TW-62 | 2020 | Gout can increase the risk of periodontal disease in Taiwan. | Chen HH |
| TW-63 | 2020 | Retrospective analysis of the association between tooth loss and dementia: a population-based matched case-control study. | Tsai YC |
| TW-64 | 2020 | Effect of Periodontitis and Scaling and Root Planing on Risk of Pharyngeal Cancer: A Nested Case-Control Study. | Chen PJ |
| TW-65 | 2020 | Increased risk of developing dental diseases in patients with primary Sjogren's syndrome-A secondary cohort analysis of population-based claims data. | Chuang CJ |
| TW-66 | 2020 | Risk of dementia in patients with periodontitis and related protective factors: A nationwide retrospective cohort study. | Lee CY |
| TW-68 | 2020 | Periodontitis and the subsequent risk of glaucoma: results from the real-world practice | Sun KT |
| TW-69 | 2021 | Bidirectional relationship between temporomandibular disorder and ankylosing spondylitis: a population-based cohort study. | Huang YF |
| TW-70 | 2021 | The impact of timing and modalities of dental prophylaxis on the risk of 5-fluorouracil-related oral mucositis in patients with head and neck cancer: a nationwide population-based cohort study. | Huang YF |
| TW-71 | 2021 | Association between obesity and chronic periodontitis: A nationwide population-based cohort study in Taiwan. | Chen TP |
| TW-72 | 2021 | Periodontitis is associated with incidental valvular heart disease: A nationwide population-based cohort study. | Sia SK |
| TW-73 | 2021 | Risk of dental malocclusion in children with upper respiratory tract disorders: A case-control study of a nationwide, population-based health claim database. | Lin SW |
| TW-74 | 2021 | Chinese Herbal Medicine to Reduce Radiation-Induced Oral Mucositis in Head and Neck Cancer Patients: Evidence From Population-Based Health Claims. | Li HH |
| TW-75 | 2021 | Coprescription Trends in Western Medicine, Chinese Herbal Medicine and Dental Medicine among Older Adults in Taiwan from 1997 to 2013 | Huang CY |
| TW-76 | 2022 | The impact of dental therapy timelines and irradiation dosages on osteoradionecrosis in oral cancer patients: A population-based cohort study. | Chang CT |
| TW-77 | 2022 | Is periodontitis a risk factor of benign or malignant colorectal tumor? A population-based cohort study. | Fu MM |
| TW-78 | 2022 | The association between the severity of periodontitis and osteonecrosis of the jaw in patients with different cancer locations: a nationwide population-based study. | Huang YF |
| TW-79 | 2025 | Risk of atopic dermatitis in periodontitis patients with and without dental scaling: A retrospective cohort study. | Chiang MC |
| TW-80 | 2025 | Association between dental scaling and the lower risk of oral cancer: A population-based nested case-control study. | Yeh YT |
| TW-81 | 2025 | The Addition of Chinese Herbal Medicines Is Effective as a Prophylactic Treatment Against Dental Diseases for Sjögren's Syndrome Patients: Insight from Real-World Database. | Juan CY |
| TW-82 | 2025 | Prior chronic periodontitis is associated with chronic rhinosinusitis: A population-based case-control study. | Yang TH |
| TW-83 | 2025 | Sjögren's syndrome increased risk of attempted suicide. | Yang TH |
| TW-84 | 2025 | A population-based study on the associations of thyroid cancer with chronic periodontitis. | Yang TH |
| TW-85 | 2024 | Dental visits and colon-rectum cancer: A nationwide population-based nested case-control study in Taiwan. | Liu FH |
| TW-86 | 2024 | Association between severity of dental caries and the risk of stroke. | Ono Y |
| TW-87 | 2023 | Increased prevalences of head and neck cancers in patients with Sjögren's syndrome. | Yang TH |
| TW-88 | 2023 | The effects of regular dental scaling on the complications and mortality after stroke: a retrospective cohort study based on a real-world database. | Sung LC |
| TW-89 | 2023 | Bidirectional Associations of Temporomandibular Joint Disorders with Major Depressive and Anxiety Disorders. | Liou YJ |
| TW-90 | 2023 | Relationship between Chinese Herbal Medicine Use and Risk of Sjögren's Syndrome in Patients with Rheumatoid Arthritis: A Retrospective, Population-Based, Nested Case-Control Study. | Liao HH |
| TW-91 | 2022 | Increased dental visits in patients with rheumatoid arthritis: a secondary cohort analysis of population based claims data. | Juan CY |
| TW-92 | 2022 | Metformin and risk of gingival/periodontal diseases in diabetes patients: A retrospective cohort study. | Tseng CH |
| TW-93 | 2022 | Risk of Peripheral Arterial Occlusive Disease with Periodontitis and Dental Scaling: A Nationwide Population-Based Cohort Study. | Yeh YT |
| TW-94 | 2022 | Stratified analysis of the association between periodontitis and female breast cancer based on age, comorbidities and level of urbanization: A population-based nested case-control study. | Chen CC |
| TW-96 | 2024 | Trends of dental service utilization and expenditure in Taiwan from 2000 to 2020 | Chen T-C |

| United Kingdom | | | |
| --- | --- | --- | --- |
| GB-1 | 2010 | The influence of economic incentives on treatment patterns in a third-party funded dental service. | Woods N |
| GB-2 | 2012 | Comparing the treatment provided by GB and non-GB trained health professionals: dentists in Scotland. | Wang S |
| GB-3 | 2012 | Ten year survival of bridges placed in the General Dental Services in England And Wales | Burke F |
| GB-4 | 2013 | Hospital care of children with a cleft in England. | Fitzsimons KJ |
| GB-5 | 2013 | Analysis of a payments database reveals trends in dental treatment provision. | Guiney H |
| GB-6 | 2014 | Hospital admissions for dental treatment among children with cleft lip and/or palate born between 1997 and 2003: an analysis of Hospital Episode Statistics in England. | Fitzsimons KJ |
| GB-7 | 2017 | Grommet Surgery in Children With Orofacial Clefts in England. | Fitzsimons KJ |
| GB-8 | 2017 | Socioeconomic disparities in orthodontic treatment outcomes and expenditure on orthodontics in England's state-funded National Health Service: a retrospective observational study. | Price J |
| GB-9 | 2019 | Frequency, variation and cost of dental extractions for adults in secondary care in Great Britain. | Hong B |
| GB-11 | 2022 | Mind the gap: an administrative data analysis of dental treatment outcomes and severe mental illness. | Ferry F |
| GB-12 | 2021 | You had to do something': prescribing antibiotics in Scotland during the COVID-19 pandemic restrictions and remobilisation | Duncan E |
| GB-13 | 2025 | Model-Based Cost-Effectiveness of Direct Restorations: Amalgam Dominates | Bailey O |
| GB-14 | 2024 | Fluoride varnish applications provided in general dental practice for children of primary school age in three areas of the UK | Brown N |
| GB-15 | 2023 | Inequalities in access to NHS primary care dental services in Scotland during the COVID-19 pandemic | Aminu AQ |
| GB-16 | 2022 | NHS general dental practitioner claims in the South West for provision of topical fluoride, fissure sealants, radiographs, fillings and extractions for children born in 2009: an analysis of a five-year period | Brown N |
| GB-17 | 2024 | How effective and cost-effective is water fluoridation for adults and adolescents? The LOTUS 10-year retrospective cohort study. | Moore D |
| GB-18 | 2025 | Investigating the Impact of Additional Congenital Malformations on Speech Outcomes at age Five in Children with a Cleft Palate. | Butterworth S |
| GB-20 | 2024 | Development of an outcome indicator framework for a universal health visiting programme using routinely collected data. | Horne M |
| GB-21 | 2025 | Cleft lip Sidedness and the Association with Additional Congenital Malformations. | Fell M |
| GB-22 | 2024 | What is the impact of the UK soft drinks industry levy on childhood tooth decay? | Watt S |
| GB-23 | 2024 | Effect of fluoridated water on invasive NHS dental treatments for adults: the LOTUS retrospective cohort study and economic evaluation. | Moore D |
| GB-25 | 2023 | Range and Frequency of Congenital Malformations Among Children With Cleft Lip and/or Palate. | Fitzsimons KJ |

| Japan | | | |
| --- | --- | --- | --- |
| JP-1 | 2010 | Relationship between community-based dental health programs and health care costs for the metabolic syndrome. (In Japanese) | Takeuchi N |
| JP-2 | 2010 | Effectiveness of Workplace Dental Checkups from a Dental Healthcare Cost Perspective. (In Japanese) | Baba M |
| JP-3 | 2011 | Survey of dental treatment expenditures based on medical expense data of dental status and general health of the elderly in Hokkaido | Yoshiuchi A |
| JP-4 | 2016 | Association between Number of Teeth and Medical and Dental Care Expenditure-Analysis Using the Receipt and Health Checkup Information Detabase in Japan- | Tsuneishi M |
| JP-5 | 2017 | Association between dental visits for periodontal treatment and type 2 diabetes mellitus in an elderly Japanese cohort. | Saito M |
| JP-7 | 2017 | Association between Number of Teeth and Medical Visit due to Aspiration Pneumonia in Older People Using the Receipt and Health Checkup Information Database. (In Japanese) | Tsuneishi M |
| JP-10 | 2018 | The Relationship Between Number of Teeth and Medical Costs in Late-Stage Elderly: Based on Dental Checkup Results and Receipt Data from the Mie Prefecture Late-Stage Elderly Medical Care Wide Area Union. (In Japanese) | Saito M |
| JP-11 | 2018 | Preoperative oral care and effect on postoperative complications after major cancer surgery. | Ishimaru M |
| JP-12 | 2019 | A Study on the Relationship Between Medical Costs, Health Status, and Oral Condition Using Big Data from a Certain Health Insurance Association. (In Japanese) | Ichihashi T |
| JP-13 | 2019 | The Presence of Teeth by Tooth Type Using the Dental Notation of Periodontitis Patients: A Cross-sectional Study Using the Receipt and Health Checkup Information Database in Japan. (In Japanese) | Tsuneishi M |
| JP-14 | 2019 | Association between perioperative oral care and postoperative pneumonia after cancer resection: conventional versus high-dimensional propensity score matching analysis. | Ishimaru M |
| JP-15 | 2020 | Differences in Annual Use of Medical and Dental Treatment and Cost among Workers of Different Business Types: Data on Insured Persons Registered with the Japan Health Insurance Association Osaka Branch. (In Japanese) | Uene M |
| JP-16 | 2020 | Evaluation of tooth loss among patients with diabetes mellitus using the National Database of Health Insurance Claims and Specific Health Checkups of Japan. | Suzuki S |
| JP-17 | 2020 | Relationship Between the Number of Occlusal Supporting and Medical Cost: Analysis Using Large Claims Database from Employee Health Care Insurance in Japan. | Murata T |
| JP-18 | 2020 | Combined Associations of Body Mass Index and Metabolic Health Status on Medical and Dental Care Days and Costs in Japanese Male Employees: A 4-Year Follow-Up Study. | Nishikawa K |
| JP-19 | 2021 | Effect of periodontal disease on the risk of development of cerebral infarction and myocardial infarction: a study using health insurance records. (In Japanese) | Ishihara T |
| JP-20 | 2021 | Relationship between Oral Health Conditions and Medical Care Expenditure Based on Receipt Information. (In Japanese) | Ishikawa M |
| JP-21 | 2021 | Long-term epidemiological study (2002–2014) on the relationship between oral health status, medical expenses, health status, and health behaviors using big data from a certain health insurance association. (In Japanese). | Ichihashi T |
| JP-22 | 2021 | Relationship Between Oral Health and Fractures in Community-Dwelling Older Japanese Adults. | Ito W |
| JP-23 | 2021 | Japan's Dental Care Facing Population Aging: How Universal Coverage Responds to the Changing Needs of the Elderly. | Okamoto E |
| JP-24 | 2021 | Association between number of teeth and Alzheimer's disease using the National Database of Health Insurance Claims and Specific Health Checkups of Japan. | Tsuneishi M |
| JP-25 | 2021 | Generic Drug Usage in Dentistry across Japan: Analysis Using a Japanese National Database. | Ono S |
| JP-26 | 2021 | Are Public Oral Care Services Evenly Distributed?-Nation-Wide Assessment of the Provision of Oral Care in Japan Using the National Database of Health Insurance Claims. | Kodama T |
| JP-27 | 2021 | Does Last Year's Cost Predict the Present Cost? An Application of Machine Leaning for the Japanese Area-Basis Public Health Insurance Database. | Nomura Y |
| JP-28 | 2021 | Validity of diagnoses and procedures in Japanese dental claims data | Ono |
| JP-29 | 2021 | Validity of congenital malformation diagnoses in healthcare claims from a university hospital in Japan | Ishikawa |
| JP-30 | 2021 | Sociodemographic inequities in dental care utilisation among governmental welfare recipients in Japan: a retrospective cohort study | Nishioka |
| JP-31 | 2022 | Trends in prophylactic antibiotic use for tooth extraction from 2015 to 2018 in Japan: An analysis using a health insurance claims database. | Sato M |
| JP-32 | 2022 | Short-term effects of reduced cost sharing on childhood dental care utilization and dental caries prevention in Japan. | Ono S |
| JP-33 | 2022 | Prevalence, Incidence Rate, and Risk Factors of Medication-Related Osteonecrosis of the Jaw in Patients With Osteoporosis and Cancer: A Nationwide Population-Based Study in Japan. | Ishimaru M |
| JP-34H | 2022 | The first national survey of antimicrobial use among dentists in Japan from 2015 to 2017 based on the national database of health insurance claims and specific health checkups of Japan | Ono A |
| JP-35H | 2021 | Effects of periodontal management for patients with type 2 diabetes on healthcare expenditure, hospitalization and worsening of diabetes: an observational study using medical, dental and pharmacy claims data in Japan | Shin JH |
| JP-36H | 2021 | Association Between Oral Health and the Medical Costs of Dementia: A Longitudinal Study of Older Japanese | Saito M |
| JP-37H | 2021 | Glycemic control and number of natural teeth: analysis of cross-sectional Japanese employment-based dental insurance claims and medical check-up data. | Harada K |
| JP-38 | 2025 | Association among the number of natural teeth, dental maintenance visits and diabetes status: a cross-sectional study using employment-based healthcare claims database | Ishikawa M |
| JP-39 | 2025 | Association between chronic diseases and severe periodontal disease progression: A retrospective cohort study in a city of Japan | Nin S |
| JP-40 | 2024 | Validity of claims-based definition of number of remaining teeth in Japan: Results from the Longevity Improvement and Fair Evidence Study | Tamada Y |
| JP-42 | 2022 | Trends in prophylactic antibiotic use for tooth extraction from 2015 to 2018 in Japan: An analysis using a health insurance claims database | Sato M |
| JP-43 | 2025 | Antibiotic Prescription Trends in Dentistry: A Descriptive Study Using Japan's National Database. | Hirayama K |
| JP-44 | 2025 | Antimicrobial prescriptions for older patients in dental medicine: A four-year retrospective study in Japan. | Kurishima A |
| JP-45 | 2024 | Longitudinal association between posterior occlusal contact and dementia development in a large retrospective cohort study using a Japanese claims database. | Miyano T |
| JP-46 | 2022 | Amoxicillin vs third-generation cephalosporin for infection prophylaxis after third molar extraction. | Sato M |
| JP-47 | 2024 | Association Between Reduced Posterior Occlusal Contact and Alzheimer's Disease Onset in Older Japanese Adults: Results from the LIFE Study. | Miyano T |
| JP-48 | 2023 | The five-year trends in antibiotic prescription by dentists and antibiotic prophylaxis for tooth extraction: a region-wide claims study in Japan. | Hirayama K |
| JP-49 | 2023 | The relationship between subjective oral frailty and adverse health outcomes or medical and dental expenditures in the latter-stage older adult: A 6-year longitudinal study. | Doi T |
| JP-50 | 2022 | Short-term effects of reduced cost sharing on childhood dental care utilization and dental caries prevention in Japan. | Ono S |
| JP-52 | 2025 | Tooth Extraction, Dental Visits, and Glucose Levels: a Retrospective Study. | Suzuki S |
| JP-53 | 2025 | Longitudinal Association Between Self-Reported Chewing Difficulty and Depression Among Older Japanese Adults Using Data From the Longevity Improvement and Fair Evidence Study. | Miyano T |
| JP-54 | 2025 | Impact of posterior occlusal contact loss on cardiovascular disease using a Japanese claims database. | Miyano T |
| JP-55 | 2025 | Differences in health care expenditure due to the comorbidity status of periodontal disease and diabetes mellitus. | Kinugawa A |
| JP-56 | 2024 | Influence of Type of Dental Visit on the Incidence of COVID-19 and Related Hospitalisation Among Older People in Japan. | Saito M |
| JP-57 | 2025 | Periodontal Care Is Associated With a Lower Risk of Dialysis Initiation in Middle-Aged Patients With Type 2 Diabetes Mellitus: A 6-Year Follow-Up Cohort Study Based on a Nationwide Healthcare Database. | Kusama T |
| JP-58 | 2025 | Risk factors for osteonecrosis of the jaw in patients with chronic kidney disease: a nested case-control study. | Iseri K |
| JP-60 | 2025 | Public Periodontal Screening Increases Subsequent Regular Dental Visits: The Life Study. | Tamada Y |
| JP-61 | 2024 | Association of oral hypofunction with aspiration pneumonia, fractures, and mortality in older Japanese adults. | Sakai K |
| JP-62 | 2024 | Predictors of Medical and Dental Clinic Closure by Machine Learning Methods: Cross-Sectional Study Using Empirical Data. | Park YT |
| JP-63 | 2025 | Association of oral frailty with medical expenditure in older Japanese adults: The study of late-stage older adults in Tottori (START Tottori). | Arai E |
| JP-64 | 2024 | Preventive dental care reduces risk of cardiovascular disease and pneumonia in hemodialysis population: a nationwide claims database analysis. | Mikami R |
| JP-65 | 2024 | The effectiveness of a self-reported questionnaire on masticatory function in health examinations. | Anzai K |
| JP-66 | 2024 | Changes in the Utilization of Outpatient and Visiting Dental Care and Per-attendance Care Cost by Age Groups During COVID-19 Pandemic Waves in Japan: A Time-series Analysis From the LIFE Study. | Kusama T |
| JP-67 | 2023 | Association between the number of teeth and incident pre-diabetes among middle-aged adults with periodontal disease: a retrospective cohort analysis of Japanese claims data. | Hasegawa K |
| JP-68 | 2024 | Are Dental Procedures Associated With Pyogenic Vertebral Osteomyelitis? | Masuda S |
| JP-69 | 2024 | Association between dental procedures and periprosthetic joint infection: A case-crossover study. | Masuda S |
| JP-70 | 2023 | Analysis of post-extraction bleeding in patients taking antithrombotic therapy using data from the longevity improvement and fair evidence study. | Nakamura J |
| JP-71 | 2023 | Dental Utilization Stratified by the Purpose of Visit: A Population-Based Study in Japan. | Ishimaru M |
| JP-72 | 2023 | Incidence and trend of antiresorptive agent-related osteonecrosis of the jaw from 2016 to 2020 in Kure, Japan. | Kunihara T |
| JP-73 | 2022 | Association between smoking habits and dental care utilization and cost using administrative claims database and specific medical check-up data. | Kawamura K |
| JP-74 | 2025 | Assessing the effectivity of counting the number of teeth with their conditions to predict mortality: the OHSAKA study | Otsuki N |
| JP-75 | 2025 | Oral Frailty, Dental Visits, and Healthy Life Expectancy: A 6-Year Prospective Cohort Among Japanese Older Adults | Khairinisa S |
| JP-77 | 2024 | Cost analysis among oral cancer patients with and without cetuximab using administrative claims database in Japan | Kitayama T |
| JP-78 | 2024 | Association between Dental Checkups at Workplaces or Dental Clinics and Expenditure or Days of Outpatient Dental Treatment: A Longitudinal Study | Mochida Y |
| JP-79 | 2023 | Investigation of Orthognathic Surgery in Japan Using the National Database | Kuwajima Y |
| JP-80 | 2023 | Association between periodontal pocket depth determined during dental check-ups and new onset of diabetes in community residents: the LIFE study | Tani N |
| JP-84 | 2025 | Association Between Dental Visits and Improvement in Self-reported Masticatory Function among Specific Health Checkup Participants: Shizuoka Kokuho Database Study | Sato Y |
| JP-87 | 2026 | Clustering Analysis of Partial Tooth Loss Patterns and Their Association With Cardiovascular Health Using a Japanese Claims Database. | Miyano T |

| Netherlands | | | |
| --- | --- | --- | --- |
| NL-1 | 2017 | Outcome measures for oral health based on clinical assessments and claims data: feasibility evaluation in practice. | Hummel R |
| NL-2 | 2019 | The differences between three performance measures on dental restorations, clinical success, survival failure: A matter of perspective. | Laske M |
| NL-3 | 2020 | Once bitten, twice shy? Lessons learned from an experiment to liberalize price regulations for dental care. | Trescher AL |
| NL-4 | 2021 | The relationship between claimed restorations, future restorations in children, adolescents: An observational follow-up study on risk categories for dental caries. | Hummel R |
| NL-5H | 2020 | Effect of periodontal treatment on diabetes-related healthcare costs: a retrospective study | Smits KPJ |
| NL-8 | 2022 | Detection of primary Sjögren's syndrome in primary care: developing a classification model with the use of routine healthcare data and machine learning. | Dros JT |
| NL-9 | 2023 | An examination of the risk of periodontitis for nonfatal cardiovascular diseases on the basis of a large insurance claims database. | Beukers NGFM |

| South Korea | | | |
| --- | --- | --- | --- |
| KR-1 | 2012 | Prevalence of birth defects in Korean livebirths, 2005-2006. | Kim MA |
| KR-2 | 2013 | A retrospective cohort study on the association between periapical abscess, advanced periodontal disease, and the national oral health screening program among Korean adults. | Ha JE |
| KR-3 | 2015 | A Large National Cohort Study of the Association between Bisphosphonates and Osteonecrosis of the Jaw in Patients with Osteoporosis: A Nested Case-control Study. | Kwon JW |
| KR-4 | 2016 | National dental policies and socio-demographic factors affecting changes in the incidence of periodontal treatments in Korean: A nationwide population-based retrospective cohort study from 2002-2013. | Lee JH |
| KR-5 | 2017 | Pediatric sialolithiasis is not related to oral or oropharyngeal infection: A population-based case control study using the Korean National Health Insurance Database. | Won SJ |
| KR-7 | 2018 | Trends in the utilization of dental outpatient services affected by the expansion of health care benefits in South Korea to include scaling: a 6-year interrupted time-series study | Park HJ |
| KR-8 | 2018 | Dose-Intensity of Bisphosphonates and the Risk of Osteonecrosis of the Jaw in Osteoporosis Patients | Jung S |
| KR-9 | 2018 | Charlson comorbidity index as a predictor of periodontal disease in elderly participants | Lee JH |
| KR-10 | 2019 | Drug holiday patterns and bisphosphonate-related osteonecrosis of the jaw. | Jung SY |
| KR-11 | 2019 | Burden of dental caries and periodontal disease in South Korea: An analysis using the national health insurance claims database. | Radnaabaatar M |
| KR-12 | 2020 | Healthcare utilisation and costs for temporomandibular disorders: a descriptive, cross-sectional study. | Seo H |
| KR-13 | 2020 | Hypertension is associated with oral, laryngeal, and esophageal cancer: a nationwide population-based study. | Seo JH |
| KR-14 | 2020 | Periodontitis combined with smoking increases risk of the ulcerative colitis: A national cohort study. | Kang EA |
| KR-15 | 2021 | Improved oral hygiene care and chronic kidney disease occurrence: A nationwide population-based retrospective cohort study. | Chang Y |
| KR-16 | 2021 | Association of Gastroesophageal Reflux Disease with Preterm Birth: Machine Learning Analysis. | Lee KS |
| KR-17 | 2021 | Risk of autoimmune diseases in recurrent aphthous ulcer patients: A nationwide population study. | Lee YC |
| KR-18 | 2021 | Uvulopalatopharyngoplasty may decrease the incidence of Parkinson's disease associated with obstructive sleep apnea. | Lee HM |
| KR-H19 | 2015 | Association of Lifestyle-Related Comorbidities With Periodontitis: A Nationwide Cohort Study in Korea | Lee JH |
| KR-H20 | 2019 | Improved oral hygiene care attenuates the cardiovascular risk of oral health disease: a population-based study from Korea | Park SY |
| KR-21 | 2024 | Association between gingivitis, tooth loss and cardiovascular risk: Insights from a 10-year nationwide cohort study of 3.7 million Koreans | Lee SY |
| KR-22 | 2023 | Analysis of the annual changes in dental institutions that claimed dental sedatives in Korea and the types of sedatives using health care big data | Lee M |
| KR-23 | 2025 | Association of Oral Health and Periodontal Disease With Oesophageal Cancer: A Nationwide Population-Based Study. | Son M |
| KR-24 | 2025 | Periodontitis and the Incidence of Inflammatory Bowel Diseases: A Nationwide Population-Based Cohort Study. | Park YMD |
| KR-25 | 2025 | Association of oral disease with cancer incidence and mortality among adults in South Korea: A nationwide retrospective cohort study. | Lee SY |
| KR-26 | 2024 | Chronic periodontal disease is related with newly developing hypertension: a nationwide cohort study. | Park JH |
| KR-27 | 2022 | Analysis of sedation and general anesthesia in patients with special needs in dentistry using the Korean healthcare big data. | Kim J |
| KR-28 | 2025 | Risk factors and incidence of oral tumors: Findings from a longitudinal population-based study. | Kim YT |
| KR-29 | 2024 | Disparity in the dental care of children and adolescents with autism spectrum disorder in Korea: a national population-based cross-sectional study. | Baek HJ |
| KR-30 | 2024 | Trend in the Incidence of Severe Partial Edentulism among Adults Using the Korean National Health Insurance Service Claim Data, 2014-2018. | Go H |
| KR-31 | 2024 | Association of periodontal disease treatment with mortality in patients with dementia: a population-based retrospective cohort study (2002-2018). | Cho HA |
| KR-32 | 2023 | Increased dental comorbidities in patients with psoriasis: a nationwide population-based cohort study in Korea. | Kwak EJ |
| KR-33 | 2023 | Association between periodontal disease status and risk of atrial fibrillation: a nationwide population-based cohort study. | Park JH |
| KR-34 | 2023 | Disparity in access for people with disabilities to outpatient dental care services: a retrospective cohort study. | Park BY |
| KR-35 | 2023 | A time-dependent subdistribution hazard model for major dental treatment events in cancer patients: a nationwide cohort study. | Han A |
| KR-36 | 2022 | Better Oral Hygiene Is Associated with a Decreased Risk of Meniere's Disease: A Nationwide Cohort Study. | Park JH |
| KR-37 | 2022 | Improved Oral Health Status Is Associated with a Lower Risk of Venous Thromboembolism: A Nationwide Cohort Study. | Park JH |
| KR-38 | 2023 | Association between periodontal disease, tooth extraction, and medication-related osteonecrosis of the jaw in women receiving bisphosphonates: A national cohort-based study. | Kwoen MJ |
| KR-39 | 2022 | The cost- effectiveness of early dental visit in infants and toddlers focused on regional deprivation in South Korea: A retrospective cohort study. | Ahn E |
| KR-40 | 2023 | Associations of Preterm Birth with Dental and Gastrointestinal Diseases: Machine Learning Analysis Using National Health Insurance Data | Song IS |

| Germany | | | |
| --- | --- | --- | --- |
| DE-1 | 2010 | Disease-specific risk for an osteonecrosis of the jaw under bisphosphonate therapy. | Jung TI |
| DE-2 | 2015 | Three-year outcomes of root canal treatment: Mining an insurance database. | Raedel M |
| DE-3 | 2015 | Three-year outcomes of apicectomy (apicoectomy): Mining an insurance database. | Raedel M |
| DE-4 | 2016 | Outcomes of direct pulp capping: interrogating and insurance database. | Raedel M |
| DE-6 | 2017 | Quality indicators for the use of systemic antibiotics in dentistry. | Hussein RJ |
| DE-7 | 2017 | Re-interventions after restoring teeth-Mining an insurance database. | Raedel M |
| DE-8 | 2017 | Four-year outcomes of restored posterior tooth surfaces-a massive data analysis | Raedel |
| DE-9 | 2019 | Tooth loss after periodontal treatment-Mining an insurance database. | Raedel M |
| DE-10 | 2020 | Comorbidity and health care utilisation in persons with Sjögren's syndrome: a claims data analysis | Albrecht K |
| DE-11 | 2020 | Prosthetic treatment patterns in the very old: an insurance database analysis from Northeast Germany. | Hempel FM |
| DE-12 | 2020 | Periodontal treatment and peripheral arterial disease severity - a retrospective analysis of health insurance claims data | Aarabi G |
| DE-13 | 2021 | Routine Data Analyses for Estimating the Caries Treatment Experience of Children. | Raedel M |
| DE-14 | 2021 | The impact of periodontal treatment on healthcare costs in newly diagnosed diabetes patients: Evidence from a German claims database. | Blaschke K |
| DE-15 | 2021 | Dental service utilization in the very old: an insurance database analysis from northeast Germany | Schwendicke F |
| DE-17 | 2025 | Utilization of dental services of older persons after onset of home care --- an observational study from the InSEMaP research project based on German insurance claims data. | Henken E |
| DE-18 | 2025 | Analysis of health insurance data on dental treatment and the occurrence of osteoradionecrosis of the jaw. | Schweyen R |
| DE-20 | 2024 | Four-year Outcomes of Class III and IV Anterior Restorations Based on a Subset of German Health Insurance Data. | Raedel M |
| DE-21 | 2024 | Detecting high-risk neighborhoods and socioeconomic determinants for common oral diseases in Germany. | Völker S |
| DE-22 | 2023 | Dental care utilization among home care recipients, nursing home residents, and older adults not in need of long-term care: An observational study based on German insurance claims data. | Czwikla J |
| DE-23 | 2022 | Associations of early childhood medication exposure and molar incisor hypomineralization (MIH): A routine data analysis. | Raedel M |
| DE-24 | 2022 | Generalizability and reach of a randomized controlled trial to improve oral health among home care recipients: comparing participants and nonparticipants at baseline and during follow-up. | Czwikla J |
| DE-25 | 2022 | Performance of fixed dental prostheses up to 6 years-A massive data analysis. | Raedel M |

| France | | | |
| --- | --- | --- | --- |
| FR-1 | 2013 | Cancer incidence estimation at a district level without a national registry: A validation study for 24 cancer sites using French health insurance and registry data. | Uhry Z |
| FR-2 | 2017 | Dental procedures, antibiotic prophylaxis, and endocarditis among people with prosthetic heart valves: nationwide population based cohort and a case crossover study. | Tubiana S |
| FR-4 | 2018 | What Are the Determinants of Dental Care Expenditures in Institutions for Adults With Disabilities? Findings From a National Survey. | Naouri D |
| FR-5 | 2019 | Evaluation of children's participation in a national dental programme in France | Bas AC |
| FR-6 | 2019 | Risks of 23 specific malformations associated with prenatal exposure to 10 antiepileptic drugs | Blotiere PO |
| FR-7 | 2020 | Impact of fees on access to dental care: evidence from France. | Bas AC |
| FR-8 | 2021 | Type 2 diabetes and its characteristics are associated with poor oral health: findings from 60,590 senior women from the E3N study | Laouali N |
| FR-9 | 2022 | Socio-Economic Determinants of Dental Service Expenditure: Findings from a French National Survey | Bas AC |
| FR-10 | 2024 | Use of free preventive dental programme for children: 10 years of Lov'U'r Teeth, France | Mascre C |
| FR-12 | 2025 | What do dentists do and earn? An exploratory analysis of French dentists with a focus on primary care teams. | Bas AC |
| FR-14 | 2022 | Hospitalizations for infections in primary Sjögren's syndrome patients: a nationwide incidence study. | Goulabchand R |

| Denmark | | | |
| --- | --- | --- | --- |
| DK-1 | 2016 | Patterns of dental services and factors that influence dental services among 64-65-year-old regular users of dental care in Denmark. | Christensen LB |
| DK-2 | 2020 | Implementation of altered provider incentives for a more individual-risk-based assignment of dental recall intervals: evidence from a health systems reform in Denmark | Gabel F |
| DK-3 | 2021 | Antimycotic Treatment of Oral Candidiasis in Warfarin Users. | Iversen DB |
| DK-4 | 2024 | Lessons learnt on patient safety in dentistry through a 5-year nationwide database study on iatrogenic harm. | Ferlias N |

| Canada | | | |
| --- | --- | --- | --- |
| CA-1 | 2014 | Trends in pediatric dental surgery for severe early childhood caries in Manitoba, Canada. | Schroth RJ |
| CA-2 | 2015 | The accuracy of International Classification of Diseases coding for dental problems not associated with trauma in a hospital emergency department | Figueiredo R |
| CA-3 | 2016 | Dental treatment and employment outcomes among social assistance recipients in Ontario, Canada. | Singhal S |
| CA-4 | 2020 | Incidence, Risk Factors, and Mortality Associated With Orofacial Cleft Among Children in Ontario, Canada. | Malic CC |
| CA-5 | 2025 | Utilization of silver diamine fluoride by dentists in Canada: a review of the Non-Insured Health Benefits Dental Claims database. | El Azrak M |
| CA-6 | 2025 | Multiple Primary Tumours in Oral Cancer: Patient Characteristics and Survival Patterns. | Sawani S |
| CA-7 | 2025 | The use of general anesthesia for dental treatment of children with special healthcare needs in Alberta, Canada. | Yazdanbakhsh E |
| CA-8 | 2024 | Healthcare Utilization of Oral and Oropharyngeal Cancer Patients in Emergency Department and Outpatient Settings: An 8-year Population-Based Study. | MiriMoghaddam M |
| CA-9 | 2024 | Trends and predictors of unplanned hospitalization among oral and oropharyngeal cancer patients; an 8-year population-based study. | MiriMoghaddam M |
| CA-10 | 2023 | The burden of psychiatric disorders associated with orofacial cleft pathology among children in Ontario, Canada. | Malic CC |
| CA-11 | 2024 | Community water fluoride cessation and rate of caries-related pediatric dental treatments under general anesthesia in Alberta, Canada | Yazdanbakhsh E |

| Australia | | | |
| --- | --- | --- | --- |
| AU-1 | 2010 | Modeling the long-term cost-effectiveness of the caries management system in an Australian population. | Warren E |
| AU-2 | 2015 | The growth of Medicare rebatable cone beam computed tomography and panoramic radiography in Australia. | Brown LF |
| AU-3 | 2020 | Use of medicines associated with dry mouth and dental visits in an Australian cohort. | Moffat AK |
| AU-4 | 2021 | Impact of COVID-19 on the provision of paediatric dental care: Analysis of the Australian Child Dental Benefits Schedule. | Hopcraft M |
| AU-6 | 2023 | Effects of the Child Dental Benefits Schedule on dental hospitalisation rates in Australian children. | Aminian P |
| AU-7 | 2025 | Dental Service Utilisation Among First Nations’ People in Southeast Queensland | Stormon N |

| Switzerland | | | |
| --- | --- | --- | --- |
| CH-1 | 2024 | Hospitalization Trends for Airway Infections and In-Hospital Complications in Cleft Lip and Palate. | Laager R |
| CH-2 | 2024 | Dental injuries in Swiss children - an analysis of Swiss national health insurance data from the year 2019. | Wieland R |
| CH-3 | 2023 | Incidence, characteristics and cost of head, neck and dental injuries in non-professional football (soccer) using 3 years of sports injury insurance data. | Hansen MG |

| Austria | | | |
| --- | --- | --- | --- |
| AT-1 | 2020 | Regional and gender differences in population-based oral health insurance data. | Stamm T |

| Finland | | | |
| --- | --- | --- | --- |
| FI-1 | 2021 | Age-related variation in volume and content of restorative private dental care for adults in Finland in 2012-2017: A nationwide register-based observation. | Vehkalahti MM |

| Italy | | | |
| --- | --- | --- | --- |
| IT-1 | 2013 | Assessing the risk of osteonecrosis of the jaw due to bisphosphonate therapy in the secondary prevention of osteoporotic fractures. | Lapi F |

| New Zealand | | | |
| --- | --- | --- | --- |
| NZ-1 | 2025 | Hospital Dental Admissions and Caries Experience Among Children With Neurodevelopmental Disabilities: A Population-Based Record Linkage Cohort Study | Schluter PJ |

**Supplementary Table 2. Standardized database groups, original database names, oldest research year, linked databases, and references.**

| Country | Database (standardized) | Original database name(s) | National/regional | Oldest research | Linked database / notes | References |
| --- | --- | --- | --- | --- | --- | --- |
| United States | Aetna Inc | Aetna Inc | National | 2009 |  | US-80 |
|  | All-Payer Claims Database | All Payer All Claims data set; Massachusetts All-Payer Claims Database | Regional | 2010 |  | US-47, US-94, US-111, US-140, US-170, US-174 |
|  | Caris Life Sciences molecular profiling | Caris Life Sciences molecular profiling | National | 2024 | Claims data | US-193 |
|  | Centers for Medicare & Medicaid Services (CMS) data | Centers for Medicare & Medicaid Services (CMS) Medicare Medicaid | National/regional | 1995 | Delta dental Children’s Health Insurance Program (CHIP) | US-1, US-4, US-5, US-6, US-7, US-8, US-9, US-11, US-13, US-14, US-16, US-17, US-20, US-24, US-25, US-26, US-27, US-28, US-30, US-34, US-35, US-37, US-38, US-39, US-40, US-41, US-43, US-45, US-46, US-48, US-49, US-50, US-52, US-53, US-54, US-56, US-57, US-59, US-62, US-65, US-66, US-67, US-70, US-73, US-77, US-78, US-79, US-84, US-86, US-90, US-91, US-92, US-99, US-102, US-104, US-110, US-112, US-118, US-123, US-127, US-135, US-137, US-142, US-145, US-146, US-147, US-148, US-149, US-151, US-156 |
|  | Community dental access program (CDP) | Community dental access program (CDP) | Regional | 2011 |  | US-55 |
|  | Consumer Directed Health Plan (CDHP) | Consumer Directed Health Plan (CDHP) | National | 2012 |  | US-63 |
|  | Delta Dental claims data | Delta Dental | Regional | 2002 |  | US-15, US-18, US-42, US-60, US-83, US-120 |
|  | Dental insurance claims data (NY State and Massachusetts) | Dental insurance claims data (NY State and Massachusetts) | Regional | 2023 |  | US-200 |
|  | Express Scripts Members | Express Scripts Members | National | 2013 |  | US-72, US-85 |
|  | HealthPartners insurance data | HealthPartners insurance data | Regional | 2023 |  | US-199 |
|  | Healthcare Cost and Utilization Project (HCUP) | The Healthcare Cost and Utilization Project (HCUP) National Inpatient Sample Kid’s Inpatient Database Nationwide Emergency Department Sample | National | 2000 | Agency for Healthcare Research and Quality | US-21, US-22, US-23, US-32, US-33, US-153 |
|  | Hospital-based database | Hospital-based database | Regional | 2010 |  | US-3, US-29, US-71, US-75, US-76, US-155 |
|  | IBM MarketScan databases | IBM Market scan; IBM MarketScan Commercial Claims and Encounters, Medicare Supplemental, and Coordination of Benefits Research Databases.; IBM MarketScan Research Databases; IBM Watson Health MarketScan; IBM Watson Medicaid MarketScan | National/regional; National | 2006 | IBM Dental Database; MEPS | US-64, US-69, US-81, US-88, US-89, US-96, US-98, US-109, US-114, US-130, US-138, US-141, US-150, US-152, US-154, US-177, US-228, US-212, US-206, US-208, US-210 |
|  | Medicaid/CHIP claims data | Children’s Health Insurance Program (CHIP); Magellan Complete Care of Florida Medicaid health plan; Alaska Medicaid claims data; Arizona Medicaid (AHCCCS) dental claims data; MassHealth (the combined Medicaid and state Children’s Health Insurance Program in Massachusetts); Medicaid (Partners for Kids, Ohio); Medicaid and CHIP claims data (6 states); Medicaid claims; Medicaid claims (Ohio); Medicaid claims (Partners For Kids, Ohio); Medicaid claims data; Medicaid data; Medicaid data from Florida; Michigan Medicaid administrative data; New York State Medicaid claims data; Oregon Health Authority and the Iowa Medicaid Program; Pennsylvania Medicaid administrative data; the Indiana Family and Social Services Administration Office of Medicaid Policy and Planning.; Virginia Medicaid dental claims; Washington State Medicaid data; Washington State Medicaid dental claims; Washington State Medicaid dental claims (3 state agencies) | Regional; National | 1998 | The original clinical trial (Paediatric Providers Against Cavities in Children's Teeth: PACT study); Electronic medical record (EMR) data; SEER | US-51, US-97, US-113, US-160, US-161, US-162, US-169, US-171, US-180, US-181, US-185, US-187, US-190, US-191, US-192, US-194, US-196, US-197, US-204, US-205, US-207, US-211, US-214, US-223, US-226 |
|  | Medicare claims data | Medicare data | Regional | 2025 | SEER (cancer registry) | US-184 |
|  | National Surgical Quality Improvement Program (NSQIP) | National Surgical Quality Improvement Program (NSQIP) | Regional | 2009 |  | US-44 |
|  | OMS National Insurance Company (OMSNIC) malpractice claims | OMS National Insurance Company (OMSNIC) malpractice claims | National | 2022 |  | US-213 |
|  | Optum Clinformatics Data Mart | Clinformatics Data Mart; Optum Clinformatics Data Mart Database | National | 2001 |  | US-124, US-182 |
|  | P&R Dental Strategies/Fluent dental claims warehouse | a commercial dental insurance claims data warehouse, P&R Dental Strategies (now known as Fluent),; a commercial dental insurance warehouse, Fluent/P&R Dental Strategies, LLC, Hamilton Township, NJ, USA, | National | 2023 |  | US-189, US-201 |
|  | PearlDiver database | Mariner private insurance claims data from the PearlDiver database | Regional | 2023 |  | US-209 |
|  | SEER Program | The Surveillance, Epidemiology, and End Results (SEER) Program | National | 1998 |  | US-58, US-61 |
|  | TRICARE health record data | TRICARE Health Record Data | National | 2010 |  | US-31 |
|  | Transformed Medicaid Statistical Information System (T-MSIS) | the 2019–2021 Transformed Medicaid Statistical Information System (T-MSIS) by CMS (22states); Transformed Medicaid Statistical Information System (T-MSIS) | Regional; National | 2022 |  | US-163, US-173, US-195 |
|  | TriNetX claims data | TriNetX claims data | National | 2025 |  | US-172 |
|  | Washington State dental/Medicaid claims data | Washington Dental Service | Regional | 2002 |  | US-2, US-19 |
|  | Wolters Kluwer Dynamic Claims Lifecycle Database | Wolters Kluwer Dynamic Claims Lifecycle Database | National | 2007 |  | US-10 |
| Taiwan | Longitudinal Health Insurance Database (LHID/LHIRD) | The Longitudinal Health Insurance Database; Longitudinal Health Insurance Database (Taiwan); Longitudinal Health Insurance Research Database 2010 (LHIRD 2010); Taiwan Longitudinal Health Insurance Database; Taiwan Longitudinal Health Insurance Database Taiwan Longitudinal Health Insurance Database 2010 (LHID2010); Taiwanese Longitudinal Health Insurance Database | National | 1996 | ・The Taiwan Cancer Registry Database ・The Registry of Catastrophic Illness database ・The Ministry of the Interior | TW-11, TW-13, TW-14, TW-16, TW-17, TW-18, TW-19, TW-20, TW-26, TW-28, TW-29, TW-30, TW-32, TW-33, TW-36, TW-37, TW-38, TW-39, TW-42, TW-43, TW-44, TW-46, TW-47, TW-48, TW-49, TW-50, TW-51, TW-52, TW-53, TW-54, TW-56, TW-59, TW-60, TW-93, TW-82, TW-85, TW-81, TW-87, TW-83, TW-84, TW-80 |
|  | Taiwan National Health Insurance Research Database (NHIRD/NHI) | The National health insurance (NHI) Research Database; Taiwan National Health Insurance (NHI) administrative data; Taiwan National Health Insurance claims data; Taiwan National Health Insurance Database; Taiwan National Health Insurance Research Database; Taiwan National Health Insurance Research Database (NHIRD); Taiwan nationwide insurance database | National | 1996 | ・The Taiwan Cancer Registry Database ・The Registry of Catastrophic Illness database | TW-1, TW-2, TW-3, TW-4, TW-5, TW-6, TW-7, TW-9, TW-10, TW-12, TW-15, TW-21, TW-22, TW-23, TW-24, TW-25, TW-27, TW-31, TW-34, TW-35, TW-40, TW-41, TW-45, TW-55, TW-57, TW-58, TW-66, TW-96, TW-88, TW-79, TW-86, TW-89, TW-92, TW-91, TW-94, TW-90 |
| Japan | DeSC health insurance claims database | DeSC health insurance claims database | Regional | 2024 | Specified Medical Checkup data | Japan: JP-52, JP-61, JP-68, JP-69 |
|  | Hospital-based database | Hospital-base database | Regional | 2012 |  | JP-28, JP-29 |
|  | JMDC Claims Database | JMDC; JMDC Claims Database | National; Regional | 2014 | Specific Health Checkup data | JP-14, JP-17, JP-31 JP-37H, Japan: JP-42, JP-45, JP-46, JP-54, JP-57, JP-77, JP-87 |
|  | Japan Gerontological Evaluation Study (JAGES) | Japan Gerontological Evaluation Study (JAGES) 2016 | National | 2025 | National long-term care insurance database | Japan: JP-75 |
|  | Kokuho Database (KDB/SKDB) | Kokuho Database (KDB); Shizuoka Kokuho Database (SKDB) | Regional | 1997 | Specific Health Checkups; dental and medical claims | JP-1, JP-2, JP-3, JP-10, JP-18, JP-27, JP-30, JP-32, JP-35H, JP-36H, Japan: JP-84 |
|  | Late Elders' Health Insurance database | Late Elders' Health Insurance database; Late Elders' Health Insurance database (Area A, Tokyo metropolitan area) | Regional | 2025 |  | Japan: JP-63, Japan: JP-44 |
|  | Longevity Improvement & Fair Evidence (LIFE) Study | Longevity Improvement & Fair Evidence (LIFE) Study | National/regional | 2023 | National Health Insurance claims; Specific Health Checkup data; Oral health screening data; Periodontal disease screening data; adult dental checkup data; National Health Insurance and Late-Stage Elderly Healthcare claims | Japan: JP-40, JP-47, JP-53, JP-60, JP-66, JP-70, JP-80 |
|  | Medical Data Vision (MDV) database | Medical Data Vision (MDV) database | Regional | 2025 |  | Japan: JP-58 |
|  | MinaCare claims database | MinaCare Co., Ltd., Japan | Regional | 2025 | Medical check-up data | Japan: JP-38 |
|  | National Database of Health Insurance Claims and Specific Health Checkups of Japan (NDB) | National Database of Health Insurance Claims and Specific Health Checkups of Japan (NDB); National Database of Health Insurance Claims of Japan (NDB) | National; National/regional | 2011 |  | JP-4, JP-7, JP-11, JP-13, JP-16, JP-23, JP-24, JP-25, JP-33, JP-34H, Japan: JP-43, JP-67, JP-79, Japan: JP-71 |
|  | National health checkup program data (Japan) | National health checkup program data (Japan) | Regional | 2024 | Health insurance claims data | Japan: JP-65 |
|  | Oral Health Screening to Assess Keys of Aging Well (OHSAKA) study | Oral Health Screening to Assess Keys of Aging Well (OHSAKA) study | Regional | 2025 | Public dental checkups; healthcare administrative/Kokuho Database (KDB) | Japan: JP-74 |
|  | Other Japanese health-insurance, claims, or checkup databases | Health Insurance corporation database; Administrative claims database and Specific Medical Check-up data (Osaka, JHIA); Company employee dental checkup and dental claims data; Health insurance administrative data (Japan); Japanese claims database; Municipal/Regional claims database | National/regional; Regional; National | 2002 | Oral health examination and medical/dental expenditure records; Oral health screening data | JP-12, JP-15, JP-20, JP-21, JP-22, Japan: JP-73, Japan: JP-78, Japan: JP-62, Japan: JP-64, Japan: JP-39, JP-48, JP-49, JP-50, JP-55, JP-56, JP-72 |
| South Korea | Health Insurance Review and Assessment (HIRA) database | Health Insurance Review and Assessment National Patient Sample; Korean Health Insurance Database (HIRA) | National | 2002 |  | KR-12, South Korea: KR-22, KR-27 |
|  | Korean National Health Insurance Service database (NHIS/KNHIS) | National Health Insurance database; Korea National Health Insurance claims; Korea National Health Insurance claims data; Korea National Health Insurance Service database; Korean Health Insurance Database; Korean National Health Insurance claims data; Korean National Health Insurance Database; Korean National Health Insurance Service (KNHIS); Korean National Health Insurance Service database; National Health Insurance Corporation claims; National Health Insurance Database Korea; National Health Insurance Database of Korea; National Health Insurance Database of Korea (NHIS); South Korea National Health Insurance database | National | 2002 | National Health Examination data; National Health Examination data; National Health Screening data; Statistics Korea death registry; National Health Screening data; Oral examination data (NHIC) | KR-1, KR-2, KR-3, KR-4, KR-5, KR-7, KR-8, KR-9, KR-10, KR-11, KR-13, KR-14, KR-15, KR-16, KR-17, KR-18, KR-H19, South Korea: KR-39, South Korea: KR-28, South Korea: KR-40, South Korea: KR-29, South Korea: KR-31, South Korea: KR-21, KR-24, South Korea: KR-30, South Korea: KR-25, South Korea: KR-32, South Korea: KR-33, South Korea: KR-23, KR-26, KR-38, South Korea: KR-34, KR-35, South Korea: KR-36 |
|  | NHIS National Health Screening Cohort (NHIS-HEALS) | The National Health Insurance System-National Health Screening Cohort (NHIS-HEALS) | National | 2002 |  | KR-H20 |
|  | NHIS National Sample Cohort | Korea National Health Insurance Service-National Sample Cohort | National | 2022 |  | South Korea: KR-37 |
| Germany | AOK health insurance claims data | Allgemeine Ortskrankenkasse (AOK) Nordost insures; AOK Saxony-Anhalt health insurance claims data | National; Regional | 2012 |  | DE-11, DE-15, Germany: DE-18 |
|  | BARMER health insurance claims data | BARMER GEK; BARMER health insurance claims data (Germany); BARMER health insurance company data warehouse (Germany) | National | 2004 |  | DE-1, DE-2, DE-3, DE-4, DE-7, DE-8, DE-9, DE-10, DE-12, DE-13, Germany: DE-23, Germany: DE-25 |
|  | BFS health finance claims data | BFS health finance claims data (Germany) | National/regional | 2024 |  | Germany: DE-20, DE-21 |
|  | German statutory health insurance claims data | The claims data of an SHI company; German statutory health and LTC insurance fund claims data (7 insurance/funds); Nationwide claims data of 8 German statutory health and LTC insurance funds; Statutory health insurance claims data (Germany) | National; Regional | 2013 |  | DE-6, Germany: DE-24, Germany: DE-22, Germany: DE-17 |
|  | InGef research database | InGef (Institute for Applied Health Research Berlin) research database | National | 2011 |  | DE-14 |
| United Kingdom | Cleft Registry and Audit NEtwork (CRANE) | Cleft Registry and Audit NEtwork (CRANE) database; UK CRANE database | National | 2023 | NHS hospital admissions data; The Hospital Episode Statistics (HES) database; NHS hospital admissions data | United Kingdom: GB-21, GB-25, United Kingdom: GB-18 |
|  | Dental Treatment Benefit Scheme / HSE data | The Health Service Executive (HSE); Dental Treatment Benefit Scheme databases | National; Regional | 1996 |  | GB-1, GB-5 |
|  | Hospital Episode Statistics / HED | Hospital Episode Statistics (HES); Secondary care NHS organizations that provide dental extractions from healthcare evaluation data (HED); Hospital Episode Statistics (HES), NHS England | National; Regional | 1997 |  | GB-4, GB-6, GB-7, GB-9, GB-22 |
|  | NHS Scotland administrative data | NHS Scotland administrative data; Routine administrative data (Scotland) | Regional | 2023 |  | GB-15, GB-20 |
|  | NHS dental claims and administrative data | Management Information and Dental Accounting System (MIDAS); The National Health Service General Dental Services (GDS); National Health Service (NHS); Dental Payment System (DPS) data; NHS Business Services Authority claims data; NHS dental claims (FP17 data); NHS dental claims data; NHS dental claims data (England); NHS dental treatment claims data (England) | Regional; National | 1990 | ・Prescribing Information System for Scotland (PRISMS) data ・An online survey of Scottish general and public health service dentists including closed and open-ended questions; Hospital-based Patient Administration System (PAS) records | GB-2, GB-12, GB-3, GB-8, GB-11, GB-16, United GB-23, GB-14, GB-13, GB-17 |
| Canada | Alberta administrative/registry data | Administrative data (publicly funded facilities, Alberta); Alberta Cancer Registry; Alberta Health Services administrative data | Regional; National | 2024 | Administrative health data | CA-6, CA-7, CA-11 |
|  | CIHI Discharge Abstract Database | Canadian Institute for Health Information (CIHI) Discharge Abstract Database | Regional | 1994 |  | CA-4 |
|  | Non-Insured Health Benefits (NIHB) claims data | Non-Insured Health Benefits (NIHB) program claims data | National | 2025 |  | CA-5 |
|  | Ontario administrative/dental public health data | The Ontario Association of Public Health Dentistry listserv; Ontario administrative health databases | Regional | 2011 | Hospital administrative data | CA-3, CA-8, CA-9, CA-10 |
|  | The Medical Claims/Physician Billings database | The Medical Claims/Physician Billings database | Regional | 1997 | The Hospital Separation Abstracts database | CA-1 |
| France | ALD long-term disease registry | ALD (Affection de Longue Dure´e) | National | 2000 | Database of the French cancer registries | FR-1 |
|  | E3N cohort | The E3N (Etude Epidémiologique auprès de femmes de l’Education Nationale) | National | 2011 | The prospective cohort study | FR-8 |
|  | French National Health Insurance / SNIIRAM-SNDS data | French National Health Insurance (SNIIRAM) database; CNAM-DGFiP dataset; French Health insurance database (nationwide); General Beneficiary Sample (medico-administrative database, France) | National | 2008 | The Health and Disability Survey Institutions Section (HSI); The French hospital discharge database (PMSI) | FR-2, FR-4, FR-6, FR-10, FR-12, FR-14 |
|  | Health and Social Protection Survey (ESPS) | The ESPS (L’Enqueˆte sante ́ et protection sociale/in French, for Health and Social Protection Survey) | National | 2006 | The Echantillon général des bénéficiaires (EGB); National health insurance data | FR-5, FR-7, FR-9 |
| Nether  lands | Achmea/Zilveren Kruis health insurance claims data | The Achmea Health Database; Claims data from Dutch healthcare insurance company Zilveren Kruis | National | 2000 | N/A | NL-1, NL-3, NL-4, NL-5H |
|  | Dental practice-based research network data | Data of General practices recruited from the Nijmegen dental practice-based research network | National |  |  | NL-2 |
|  | Dutch health insurance claims data | Healthcare insurance claims database (Netherlands); Netherlands health insurance claims database | Regional | 2022 |  | NL-8, NL-9 |
| Australia | Department of Veterans' Affairs (DVA) data | The Australian Government Department of Veterans’ Affairs (DVA) | National | 2016 |  | AU-3 |
|  | Medical Benefits Fund (MBF) data | Medical Benefits Fund (MBF) from the second largest private health insurer in Australia | National | 2007 |  | AU-1 |
|  | Medicare / Child Dental Benefits Schedule (CDBS) data | Medicare dataset (aggregated); Medicare data on utilization of the Child Dental Benefits Schedule (CDBS) (aggregated); Medicare/CDBS claims | National | 2011 |  | AU-2, AU-4, AU-6 |
|  | Queensland Health administrative dental data | Queensland Health administrative dental data | Regional | 2025 |  | AU-7 |
| Denmark | Danish Dental Compensation Act (DDCA) database | Danish Dental Compensation Act (DDCA) database | National | 2024 |  | DK-4 |
|  | Danish National Health Insurance database | The Danish National Health Insurance database | National | 2009 | Statistics Denmark | DK-1, DK-2 |
|  | The Copenhagen Primary Care Laboratory (CopLab) database | The Copenhagen Primary Care Laboratory (CopLab) database | National | 2000 |  | DK-3 |
| Switzer  land | Concordia (Swiss health insurer) accident claims data | Concordia (Swiss health insurer) accident claims data | National | 2024 |  | CH-2 |
|  | National Inpatient Sample | National Inpatient Sample | National | 2024 |  | CH-1 |
|  | Swiss sports injury insurance claims database | Swiss sports injury insurance claims database | National | 2023 |  | CH-3 |
| Austria | Population-based health insurance data | Population-based health insurance data | National | 2012 |  | AT-1 |
| Finland | The Social Insurance Institute database of private dental care services (aggregated) | The Social Insurance Institute database of private dental care services (aggregated) | National | 2012 |  | FI-1 |
| Italy | Bisphosphonates Effectiveness Safety Trade-off (BEST) network | Bisphosphonates Effectiveness Safety Trade-off (BEST) network | Regional | 2003 |  | IT-1 |
| New Zealand | National administrative health data (New Zealand) | National administrative health data (New Zealand) | National | 2025 |  | NZ-1 |

Note: Original database names were consolidated into standardized database groups to account for variation in terminology across studies.

**Supplementary Table 3. Study designs of included studies with reference IDs.**

| Study design | n (%) | references |
| --- | --- | --- |
| Cohort study | 226 (47.9) | **Japan:** JP-11, JP-14, JP-17, JP-18, JP-19, JP-22, JP-27, JP-30, JP-33, JP-35H, JP-36H, JP-39, JP-45, JP-46, JP-47, JP-49, JP-50, JP-52, JP-53, JP-54, JP-55, JP-56, JP-57, JP-60, JP-61, JP-64, JP-67, JP-74, JP-75, JP-77, JP-78, JP-80, JP-84, JP-87  **United States:** US-1, US-6, US-8, US-10, US-12, US-13, US-14, US-16, US-17, US-25, US-27, US-28, US-36, US-40, US-49, US-50, US-51, US-52, US-56, US-58, US-61, US-62, US-64, US-65, US-69, US-76, US-77, US-78, US-79, US-80, US-81, US-83, US-84, US-85, US-89, US-90, US-94, US-95, US-96, US-110, US-112, US-113, US-120, US-123, US-124, US-126, US-130, US-132, US-135, US-136, US-141, US-146, US-147, US-152, US-153, US-156, US-157, US-158, US-161, US-162, US-169, US-171, US-172, US-177, US-181, US-187, US-189, US-190, US-192, US-193, US-194, US-199, US-200, US-209, US-211, US-214, US-228  **United Kingdom:** GB-2, GB-3, GB-4, GB-17, GB-23  **South Korea:** KR-2, KR-4, KR-8, KR-9, KR-13, KR-14, KR-15, KR-16, KR-17, KR-18, KR-H19, KR-H20, KR-21, KR-24, KR-25, KR-26, KR-28, KR-31, KR-32, KR-33, KR-34, KR-35, KR-36, KR-37, KR-38, KR-39, KR-40  **Taiwan:** TW-2, TW-3, TW-4, TW-9, TW-10, TW-14, TW-15, TW-16, TW-17, TW-20, TW-22, TW-23, TW-24, TW-28, TW-31, TW-32, TW-36, TW-37, TW-38, TW-39, TW-43, TW-44, TW-45, TW-48, TW-49, TW-50, TW-51, TW-54, TW-55, TW-56, TW-59, TW-61, TW-62, TW-65, TW-66, TW-68, TW-69, TW-71, TW-72, TW-74, TW-76, TW-77, TW-79, TW-83, TW-86, TW-88, TW-89, TW-91, TW-92, TW-93  **Germany:** DE-2, DE-3, DE-4, DE-7, DE-8, DE-9, DE-11, DE-12, DE-13, DE-14, DE-15, DE-17, DE-18, DE-20, DE-24, DE-25  **Netherlands:** NL-4, NL-5H, NL-9  **France:** FR-2, FR-6, FR-14  **Denmark:** DK-3  **Canada:** CA-3, CA-4, CA-6, CA-7, CA-8, CA-9, CA-10, CA-11  **Switzerland:** CH-1  **New Zealand:** NZ-1 |
| Cross-sectional study | 109 (23.1) | **Japan:** JP-2, JP-3, JP-4, JP-5, JP-7, JP-10, JP-12, JP-13, JP-20, JP-21, JP-23, JP-24, JP-37H, JP-38, JP-43, JP-44, JP-48, JP-62, JP-63, JP-65, JP-70, JP-71, JP-73  **United States:** US-2, US-4, US-7, US-9, US-18, US-19, US-20, US-21, US-26, US-29, US-30, US-31, US-32, US-33, US-35, US-37, US-39, US-41, US-42, US-43, US-44, US-45, US-46, US-47, US-48, US-53, US-54, US-57, US-59, US-60, US-63, US-67, US-70, US-71, US-72, US-73, US-75, US-86, US-91, US-92, US-104, US-114, US-116, US-118, US-131, US-140, US-148, US-149, US-151, US-154, US-163, US-174, US-180, US-184, US-185, US-191, US-195, US-197, US-201, US-206, US-207, US-208, US-212, US-213, US-226  **United Kingdom:** GB-14  **South Korea:** KR-10, KR-12, KR-29, KR-30  **Taiwan:** TW-11, TW-13, TW-18, TW-29, TW-34, TW-35, TW-70, TW-87  **Germany:** DE-10, DE-22  **France:** FR-4, FR-5, FR-8, FR-9  **Denmark:** DK-2  **Australia:** AU-7 |
| Descriptive analysis | 77 (16.3) | **Japan:** JP-1, JP-15, JP-16, JP-28, JP-29, JP-31, JP-34H, JP-42, JP-72, JP-79  **United States:** US-3, US-22, US-23, US-24, US-88, US-97, US-98, US-99, US-102, US-109, US-129, US-133, US-137, US-138, US-142, US-145, US-150, US-155, US-170, US-173, US-204, US-205, US-210, US-223  **United Kingdom:** GB-5, GB-6, GB-7, GB-8, GB-9, GB-11, GB-12, GB-13, GB-15, GB-16, GB-18, GB-20, GB-21, GB-25  **South Korea:** KR-1, KR-22, KR-27  **Taiwan:** TW-1, TW-21, TW-25, TW-27, TW-57, TW-58, TW-75, TW-96  **Germany:** DE-1, DE-6  **Netherlands:** NL-3  **France:** FR-7, FR-10, FR-12  **Denmark:** DK-1, DK-4  **Australia:** AU-2, AU-3, AU-4  **Canada:** CA-1, CA-2, CA-5  **Switzerland:** CH-2, CH-3  **Finland:** FI-1  **Austria:** AT-1 |
| Case-control study | 36 (7.6) | **Japan:** JP-58  **United States:** US-5, US-34, US-127, US-182, US-196  **South Korea:** KR-3, KR-5, KR-23  **Taiwan:** TW-5, TW-6, TW-7, TW-26, TW-30, TW-33, TW-40, TW-41, TW-42, TW-47, TW-52, TW-53, TW-60, TW-63, TW-64, TW-73, TW-78, TW-80, TW-81, TW-82, TW-84, TW-85, TW-90, TW-94  **Germany:** DE-23  **Netherlands:** NL-8  **Italy:** IT-1 |
| Ecological study | 8 (1.7) | **Japan:** JP-25, JP-26, JP-32, JP-66  **United Kingdom:** GB-1  **Germany:** DE-21  **France:** FR-1  **Australia:** AU-6 |
| Interrupted time-series study | 4 (0.8) | **United States:** US-55, US-111  **United Kingdom:** GB-22  **South Korea:** KR-7 |
| Validation study | 4 (0.8) | **Japan:** JP-40  **United States:** US-66  **Netherlands:** NL-1, NL-2 |
| Simulation study | 1 (0.2) | **Australia:** AU-1 |
| Others^†^ | 7 (1.5) | **Japan:** JP-68, JP-69  **United States:** US-160  **South Korea:** KR-11  **Taiwan:**TW-12, TW-19, TW-46 |

^†^Others include publications that are difficult to categorize, such as those using original research designs or not clearly indicating their research design.

**Supplementary Table 4. Grouped exposure and outcome variables in included studies with reference IDs.**

| Oral health / dental practice category | Systemic health association | n | References |
| --- | --- | --- | --- |
| Dental visit/procedure | Total | 107 | **United States:** US-1, US-3, US-6, US-7, US-9, US-13, US-14, US-18, US-19, US-20, US-21, US-24, US-26, US-27, US-30, US-33, US-40, US-42, US-45, US-47, US-48, US-51, US-52, US-53, US-55, US-59, US-60, US-65, US-66, US-67, US-70, US-71, US-73, US-76, US-91, US-109, US-111, US-112, US-113, US-116, US-120, US-131, US-135, US-136, US-137, US-138, US-140, US-145, US-149, US-150, US-157, US-160, US-161, US-162, US-163, US-169, US-171, US-177, US-181, US-191, US-195, US-197, US-200, US-204, US-205, US-208, US-210, US-211, US-223, US-226, US-228  **Taiwan:** TW-19, TW-29, TW-34, TW-61, TW-85, TW-91, TW-96  **United Kingdom:** GB-4, GB-5, GB-6, GB-8, GB-9, GB-11, GB-15  **Japan:** JP-26, JP-30  **South Korea:** KR-7, KR-12, KR-35, KR-36, KR-37  **Germany:** DE-10, DE-11, DE-15, DE-17, DE-24  **France:** FR-2, FR-9  **Denmark:** DK-1, DK-2  **Canada:** CA-3, CA-7  **Australia:** AU-3, AU-4, AU-7  **Austria:** AT-1 |
|  | Cancer | 2 | **Taiwan:** TW-85  **South Korea:** KR-35 |
|  | Cardiovascular disease | 3 | **United States:** US-228  **South Korea:** KR-37  **France:** FR-2 |
|  | Diabetes mellitus | 1 | **Taiwan:** TW-29 |
|  | Health care cost | 1 | **South Korea:** KR-12 |
|  | Pregnancy | 2 | **United States:** US-161, US-195 |
|  | Rheumatoid arthritis | 1 | **Taiwan:** TW-91 |
|  | Others | 3 | **United States:** US-177, US-211  **South Korea:** KR-36 |
| Oral and maxillofacial surgery | Total | 82 | **United States:** US-10, US-17, US-22, US-23, US-28, US-35, US-43, US-44, US-50, US-56, US-58, US-64, US-84, US-92, US-98, US-104, US-124, US-184, US-212, US-213  **Taiwan:** TW-1, TW-2, TW-3, TW-4, TW-6, TW-10, TW-11, TW-13, TW-14, TW-21, TW-24, TW-28, TW-31, TW-42, TW-44, TW-46, TW-50, TW-51, TW-52, TW-53, TW-54, TW-55, TW-57, TW-65, TW-69, TW-74, TW-76, TW-78, TW-89  **United Kingdom:** GB-4, GB-6, GB-7, GB-18, GB-21, GB-25  **Japan:** JP-11, JP-14, JP-31, JP-33, JP-34H  **South Korea:** KR-1, KR-3, KR-5, KR-8, KR-10, KR-12, KR-13, KR-18, KR-28  **Germany:** DE-1, DE-18  **France:** FR-1, FR-2  **Denmark:** DK-3  **Canada:** CA-4, CA-6, CA-8, CA-9, CA-10  **Australia:** AU-3  **Italy:** IT-1  **Switzerland:** CH-1 |
|  | Cancer | 2 | **Canada:** CA-8, CA-9 |
|  | Cardiovascular disease | 3 | **Taiwan:** TW-46, TW-50; France: FR-2 |
|  | Health care cost | 1 | **South Korea:** KR-12 |
|  | Osteonecrosis of the jaw | 10 | **Taiwan:** TW-53, TW-55, TW-78  **Japan:** JP-33  **South Korea:** KR-3, KR-8, KR-10  **Germany:** DE-1, DE-18  **Italy:** IT-1 |
|  | Osteoporosis | 2 | **Taiwan:** TW-51, TW-54 |
|  | Respiratory disease | 5 | **United States:** US-50  **Japan:** JP-11, JP-14  **South Korea:** KR-18  **Switzerland:** CH-1 |
|  | Others | 6 | **United States:** US-92, US-212  **Taiwan:** TW-31, TW-89  **Denmark:** DK-3  **Canada:** CA-10 |
| Periodontal disease | Total | 76 | **United States:** US-2, US-12, US-29, US-36, US-69, US-80, US-132, US-146, US-158, US-199, US-206  **Taiwan:** TW-5, TW-7, TW-9, TW-12, TW-15, TW-16, TW-17, TW-23, TW-32, TW-33, TW-35, TW-36, TW-37, TW-39, TW-40, TW-41, TW-43, TW-47, TW-48, TW-49, TW-56, TW-60, TW-62, TW-64, TW-66, TW-68, TW-71, TW-72, TW-77, TW-78, TW-79, TW-80, TW-82, TW-84, TW-88, TW-92, TW-93, TW-94  **Japan:** JP-5, JP-13, JP-19, JP-35H  **South Korea:** KR-2, KR-4, KR-9, KR-11, KR-14, KR-15, KR-H19, KR-H20, KR-21, KR-23, KR-24, KR-25, KR-26, KR-31, KR-32, KR-33, KR-38  **Germany:** DE-9, DE-12, DE-14  **Netherlands:** NL-5H, NL-9  **France:** FR-8 |
|  | Allergic rhinitis | 2 | **Taiwan:** TW-32, TW-33 |
|  | Cancer | 7 | **Taiwan:** TW-12, TW-64, TW-80, TW-84, TW-94  **South Korea:** KR-23, KR-25 |
|  | Cardiovascular disease | 8 | **United States:** US-199  **Taiwan:** TW-72, TW-93  **Japan:** JP-19  **South Korea:** KR-H20, KR-33  **Germany:** DE-12  **Netherlands:** NL-9 |
|  | Cerebrovascular disease | 1 | **Japan:** JP-19 |
|  | Dementia | 3 | **United States:** US-146  **Taiwan:** TW-66  **South Korea:** KR-31 |
|  | Diabetes mellitus | 8 | **United States:** US-2, US-199, US-206  **Taiwan:** TW-92  **Japan:** JP-5, JP-35H  **Netherlands:** NL-5H  **France:** FR-8 |
|  | Health care cost | 3 | **United States:** US-69, US-199  **Germany:** DE-14 |
|  | Hypertension | 1 | **South Korea:** KR-26 |
|  | Kidney disease | 2 | **Taiwan:** TW-16  **South Korea:** KR-15 |
|  | Osteonecrosis of the jaw | 2 | **Taiwan:** TW-78  **South Korea:** KR-38 |
|  | Osteoporosis | 3 | **Taiwan:** TW-17, TW-35, TW-39 |
|  | Pregnancy | 3 | **United States:** US-12, US-80, US-132 |
|  | Respiratory disease | 4 | **Taiwan:** TW-23, TW-36, TW-37, TW-43 |
|  | Rheumatoid arthritis | 4 | **Taiwan:** TW-7, TW-9, TW-15, TW-40 |
|  | Others | 20 | **United States:** US-158  **Taiwan:** TW-5, TW-41, TW-47, TW-48, TW-49, TW-56, TW-60, TW-62, TW-68, TW-71, TW-77, TW-79, TW-88  **South Korea:** KR-2, KR-9, KR-14, KR-H19, KR-24, KR-32 |
| Dental caries/preventive care | Total | 64 | **United States:** US-4, US-25, US-37, US-42, US-49, US-51, US-54, US-60, US-62, US-63, US-65, US-78, US-83, US-96, US-97, US-102, US-110, US-118, US-123, US-127, US-138, US-141, US-142, US-147, US-148, US-151, US-152, US-170, US-173, US-174, US-181, US-185, US-187, US-189, US-190, US-194, US-201, US-207, US-214  **Taiwan:** TW-20, TW-27, TW-29, TW-38, TW-45, TW-70, TW-86  **United Kingdom:** GB-13, GB-16; South Korea: KR-11  **Germany:** DE-2, DE-3, DE-4, DE-8, DE-13, DE-20, DE-23  **Netherlands:** NL-2, NL-4  **France:** FR-10  **Canada:** CA-1, CA-5, CA-11  **Finland:** FI-1  **New Zealand:** NZ-1 |
|  | Allergic rhinitis | 1 | **Taiwan:** TW-45 |
|  | Cardiovascular disease | 1 | **Taiwan:** TW-20 |
|  | Diabetes mellitus | 1 | **Taiwan:** TW-29 |
|  | Health care cost | 1 | **United States:** US-194 |
|  | Osteonecrosis of the jaw | 1 | **Taiwan:** TW-38 |
|  | Others | 3 | **Taiwan:** TW-70, TW-86  **New Zealand:** NZ-1 |
| Prosthesis | Total | 5 | **United States:** US-39, US-153, US-209  **Japan:** JP-87  **Germany:** DE-25 |
|  | Cardiovascular disease | 1 | **Japan:** JP-87 |
|  | Others | 1 | **United States:** US-209 |
| Cost | Total | 39 | **United States:** US-17, US-25, US-36, US-37, US-47, US-54, US-69, US-77, US-78, US-90, US-127, US-136, US-147, US-148, US-151, US-152, US-154, US-180  **Taiwan:** TW-96  **United Kingdom:** GB-5, GB-13  **Japan:** JP-1, JP-2, JP-3, JP-4, JP-10, JP-12, JP-17, JP-20, JP-21, JP-27, JP-36H  **South Korea:** KR-39  **Germany:** DE-14  **Netherlands:** NL-3  **France:** FR-4, FR-9  **Australia:** AU-1  **Switzerland:** CH-3 |
|  | Health care cost | 2 | **United States:** US-69  **Germany:** DE-14 |
| Health policy/program | Total | 40 | **United States:** US-4, US-8, US-16, US-30, US-45, US-57, US-62, US-76, US-79, US-83, US-96, US-102, US-111, US-112, US-118, US-142, US-155, US-192  **United Kingdom:** GB-1, GB-2, GB-3, GB-8, GB-14, GB-17, GB-20, GB-22, GB-23  **Japan:** JP-1, JP-2, JP-32  **South Korea:** KR-4, KR-7, KR-34  **Germany:** DE-6  **France:** FR-5, FR-7, FR-12  **Denmark:** DK-2, DK-4  **Australia:** AU-6 |
|  | Cancer | 1 | **United States:** US-192 |
| Others | Total | 68 | **United States:** US-31, US-34, US-41, US-46, US-72, US-75, US-81, US-85, US-86, US-88, US-89, US-92, US-94, US-95, US-97, US-99, US-114, US-126, US-130, US-155, US-156, US-172, US-196  **Taiwan:** TW-18, TW-22, TW-25, TW-26, TW-30, TW-31, TW-57, TW-58, TW-59, TW-63, TW-73, TW-75, TW-81, TW-83, TW-87, TW-90  **United Kingdom:** GB-2, GB-12  **Japan:** JP-12, JP-13, JP-16, JP-22, JP-23, JP-24, JP-25, JP-26, JP-28, JP-31, JP-34H, JP-37H  **South Korea:** KR-16, KR-17, KR-22, KR-27, KR-29, KR-30  **Germany:** DE-7  **Netherlands:** NL-1, NL-8  **France:** FR-6, FR-14  **Canada:** CA-2, CA-3  **Australia:** AU-2  **Switzerland:** CH-2 |
|  | Cancer | 2 | **United States:** US-196  **Taiwan:** TW-87 |
|  | Rheumatoid arthritis | 1 | **Taiwan:** TW-90 |
|  | Others | 4 | **United States:** US-92, US-172  **Taiwan:** TW-31, TW-81 |

**Note:** Rows labelled Total indicate all studies in the corresponding oral/dental category. Rows listing systemic health associations are non-mutually exclusive subcategories and therefore do not sum to the Total.

Within each category, Others is listed last regardless of count.

**Supplementary Table 5. Study-level Newcastle-Ottawa Scale risk-of-bias ratings.**

| Country | Study ID | Author | Year | Study design | Selection | Comparability | Outcome/Exposure | Total NOS | Overall risk |
| --- | --- | --- | --- | --- | --- | --- | --- | --- | --- |
| Japan | JP-11 | Ishimaru et al. | 2018 | Retrospective observational cohort study | 4 | 2 | 2 | 8 | Low risk |
| Japan | JP-14 | Ishimaru et al. | 2019 | Retrospective observational cohort study | 3 | 2 | 2 | 7 | Low risk |
| Japan | JP-17 | Murata et al. | 2020 | Retrospective claims-database cohort/association study, with temporality caution | 3 | 1 | 3 | 7 | Low risk |
| Japan | JP-18 | Nishikawa & Yamamoto | 2020 | Cohort study | 4 | 2 | 3 | 9 | Low risk |
| Japan | JP-19 | Ishihara et al. | 2021 | Cohort study | 4 | 2 | 2 | 8 | Low risk |
| Japan | JP-22 | Ito et al. | 2021 | Cohort study | 3 | 2 | 3 | 8 | Low risk |
| Japan | JP-27 | Nomura et al. | 2021 | Retrospective cohort-style claims database study / predictive modeling study | 3 | 1 | 2 | 6 | Moderate risk |
| Japan | JP-30 | Nishioka et al. | 2020 | Cohort study | 4 | 2 | 2 | 8 | Low risk |
| Japan | JP-33 | Ishimaru et al. | 2022 | Retrospective cohort study | 4 | 2 | 2 | 8 | Low risk |
| Japan | JP-35H | Shin et al. | 2021 | Retrospective cohort study | 4 | 2 | 2 | 8 | Low risk |
| Japan | JP-36H | Saito et al. | 2021 | Cohort study | 4 | 1 | 2 | 7 | Low risk |
| Japan | JP-39 | Nin et al. | 2024 | Retrospective cohort study | 4 | 2 | 2 | 8 | Low risk |
| Japan | JP-40 | Tamada et al. | 2024 | Retrospective cohort component, with validation-study component noted separately | 4 | 1 | 2 | 7 | Low risk |
| Japan | JP-45 | Miyano et al. | 2024 | Retrospective cohort study | 4 | 2 | 2 | 8 | Low risk |
| Japan | JP-46 | Sato et al. | 2022 | Retrospective cohort study with propensity-score matching | 4 | 2 | 2 | 8 | Low risk |
| Japan | JP-47 | Miyano et al. | 2023 | Retrospective cohort study | 4 | 2 | 2 | 8 | Low risk |
| Japan | JP-49 | Doi et al. | 2023 | Cohort study | 4 | 1 | 3 | 8 | Low risk |
| Japan | JP-50 | Ono et al. | 2022 | Cohort study | 4 | 1 | 3 | 8 | Low risk |
| Japan | JP-52 | Suzuki et al. | 2025 | Retrospective cohort study | 3 | 1 | 3 | 7 | Low risk |
| Japan | JP-53 | Miyano et al. | 2025 | Retrospective cohort study | 4 | 1 | 2 | 7 | Low risk |
| Japan | JP-54 | Miyano et al. | 2025 | Retrospective cohort study | 4 | 2 | 2 | 8 | Low risk |
| Japan | JP-55 | Kinugawa et al. | 2025 | Retrospective cohort study | 3 | 1 | 3 | 7 | Low risk |
| Japan | JP-56 | Saito et al. | 2024 | Retrospective cohort study | 4 | 1 | 2 | 7 | Low risk |
| Japan | JP-57 | Kusama et al. | 2025 | Retrospective cohort study | 4 | 2 | 2 | 8 | Low risk |
| Japan | JP-58 | Iseri & Hida | 2024 | Nested case-control study | 3 | 2 | 3 | 8 | Low risk |
| Japan | JP-60 | Tamada et al. | 2025 | Cohort study | 3 | 2 | 3 | 8 | Low risk |
| Japan | JP-61 | Sakai et al. | 2024 | Retrospective cohort study | 3 | 1 | 3 | 7 | Low risk |
| Japan | JP-64 | Mikami et al. | 2024 | Retrospective cohort study | 4 | 2 | 2 | 8 | Low risk |
| Japan | JP-67 | Hasegawa et al. | 2023 | Retrospective cohort study | 4 | 1 | 2 | 7 | Low risk |
| Japan | JP-74 | Otsuki et al. | 2025 | Retrospective cohort study | 4 | 2 | 2 | 8 | Low risk |
| Japan | JP-75 | Khairinisa et al. | 2025 | Prospective cohort study | 4 | 2 | 2 | 8 | Low risk |
| Japan | JP-77 | Kitayama et al. | 2024 | Retrospective cohort study | 3 | 1 | 2 | 6 | Moderate risk |
| Japan | JP-78 | Mochida et al. | 2024 | Retrospective longitudinal cohort study | 2 | 1 | 3 | 6 | Moderate risk |
| Japan | JP-80 | Tani et al. | 2023 | Retrospective cohort study | 4 | 1 | 2 | 7 | Low risk |
| Japan | JP-84 | Sato et al. | 2025 | Retrospective cohort study | 4 | 2 | 2 | 8 | Low risk |
| Japan | JP-87 | Miyano et al. | 2025 | Retrospective cohort study | 3 | 2 | 2 | 7 | Low risk |
| United States | US-5 | Skaar, et al. | 2011 | Case-control study | 3 | 1 | 2 | 6 | Moderate risk |
| United States | US-6 | Chi, et al. | 2011 | Cohort study | 3 | 1 | 3 | 7 | Low risk |
| United States | US-8 | Wallace, et al. | 2011 | Cohort study | 3 | 1 | 2 | 6 | Moderate risk |
| United States | US-10 | Streeter, et al. | 2011 | Cohort study | 4 | 1 | 2 | 7 | Low risk |
| United States | US-12 | Albert, et al. | 2011 | Cohort study | 4 | 1 | 3 | 8 | Low risk |
| United States | US-13 | Kenney, et al. | 2011 | Cohort study | 2 | 1 | 2 | 5 | Moderate risk |
| United States | US-14 | Skaar, et al. | 2012 | Cohort study | 4 | 1 | 2 | 7 | Low risk |
| United States | US-16 | Stearns, et al. | 2012 | Cohort study | 4 | 1 | 3 | 8 | Low risk |
| United States | US-17 | Jacobson, et al. | 2012 | Cohort study | 4 | 1 | 2 | 7 | Low risk |
| United States | US-27 | Kuthy, et al. | 2013 | Cohort study | 3 | 1 | 3 | 7 | Low risk |
| United States | US-28 | Beadle, et al. | 2013 | Cohort study | 4 | 1 | 2 | 7 | Low risk |
| United States | US-36 | Jeffcoat, et al. | 2014 | Cohort study | 3 | 1 | 2 | 6 | Moderate risk |
| United States | US-40 | Kuthy, et al. | 2014 | Cohort study | 2 | 1 | 3 | 6 | Moderate risk |
| United States | US-49 | Herndon, et al. | 2015 | Cohort study | 3 | 1 | 3 | 7 | Low risk |
| United States | US-50 | Xu, et al. | 2015 | Cohort study | 4 | 1 | 3 | 8 | Low risk |
| United States | US-51 | Sen, et al. | 2016 | Cohort study | 3 | 1 | 3 | 7 | Low risk |
| United States | US-52 | Singhal, et al. | 2016 | Cohort study | 4 | 1 | 2 | 7 | Low risk |
| United States | US-56 | Tanvetyanon, et al. | 2016 | Cohort study | 4 | 1 | 3 | 8 | Low risk |
| United States | US-58 | Patel, et al. | 2016 | Cohort study | 4 | 1 | 2 | 7 | Low risk |
| United States | US-61 | Lemieux, et al. | 2016 | Cohort study | 4 | 1 | 2 | 7 | Low risk |
| United States | US-62 | Veschusio, et al. | 2016 | Cohort study | 2 | 1 | 2 | 5 | Moderate risk |
| United States | US-64 | Brietzke, et al. | 2017 | Cohort study | 4 | 1 | 2 | 7 | Low risk |
| United States | US-65 | Blackburn, et al. | 2017 | Cohort study | 3 | 1 | 2 | 6 | Moderate risk |
| United States | US-69 | Nasseh, et al. | 2017 | Cohort study | 4 | 1 | 3 | 8 | Low risk |
| United States | US-76 | Quinonez, et al. | 2018 | Cohort study | 2 | 1 | 3 | 6 | Moderate risk |
| United States | US-78 | Pourat, et al. | 2018 | Cohort study | 4 | 1 | 2 | 7 | Low risk |
| United States | US-79 | Park, et al. | 2018 | Cohort study | 4 | 1 | 3 | 8 | Low risk |
| United States | US-80 | Ananth, et al. | 2018 | Cohort study | 3 | 1 | 3 | 7 | Low risk |
| United States | US-81 | Harbaugh, et al. | 2018 | Cohort study | 4 | 1 | 3 | 8 | Low risk |
| United States | US-83 | Huang, et al. | 2018 | Cohort study | 3 | 1 | 3 | 7 | Low risk |
| United States | US-84 | Xiang, et al. | 2018 | Cohort study | 4 | 1 | 3 | 8 | Low risk |
| United States | US-89 | Suda, et al. | 2019 | Cohort study | 3 | 1 | 3 | 7 | Low risk |
| United States | US-94 | Ranade, et al. | 2019 | Cohort study | 4 | 1 | 3 | 8 | Low risk |
| United States | US-95 | Shelton, et al. | 2019 | Cohort study | 3 | 1 | 1 | 5 | Moderate risk |
| United States | US-96 | Tiwari, et al. | 2019 | Cohort study | 2 | 1 | 2 | 5 | Moderate risk |
| United States | US-110 | Okunseri, et al. | 2020 | Cohort study | 3 | 1 | 3 | 7 | Low risk |
| United States | US-112 | Song, et al. | 2020 | Cohort study | 3 | 1 | 2 | 6 | Moderate risk |
| United States | US-113 | Stockbridge, et al. | 2020 | Cohort study | 2 | 1 | 2 | 5 | Moderate risk |
| United States | US-120 | Yavorek, et al. | 2020 | Cohort study | 4 | 1 | 3 | 8 | Low risk |
| United States | US-124 | Makar, et al. | 2020 | Cohort study | 4 | 1 | 2 | 7 | Low risk |
| United States | US-126 | Yepes, et al. | 2020 | Cohort study | 4 | 1 | 2 | 7 | Low risk |
| United States | US-130 | Ahmed, et al. | 2021 | Cohort study | 4 | 1 | 2 | 7 | Low risk |
| United States | US-132 | Choi, et al. | 2021 | Cohort study | 3 | 1 | 3 | 7 | Low risk |
| United States | US-135 | Kranz, et al. | 2021 | Cohort study | 3 | 1 | 2 | 6 | Moderate risk |
| United States | US-136 | Choi, et al. | 2021 | Cohort study | 3 | 1 | 2 | 6 | Moderate risk |
| United States | US-141 | McGivern, et al. | 2021 | Cohort study | 4 | 1 | 2 | 7 | Low risk |
| United States | US-146 | Malone, et al. | 2022 | Cohort study | 4 | 1 | 2 | 7 | Low risk |
| United States | US-147 | Taylor, et al. | 2022 | Cohort study | 3 | 1 | 3 | 7 | Low risk |
| United States | US-152 | Okunev, et al. | 2022 | Cohort study | 3 | 1 | 3 | 7 | Low risk |
| United States | US-153 | Choi, et al. | 2022 | Cohort study | 3 | 1 | 3 | 7 | Low risk |
| United States | US-156 | Khouja, et al. | 2022 | Cohort study | 4 | 1 | 3 | 8 | Low risk |
| United States | US-157 | Choi, et al. | 2022 | Cohort study | 3 | 1 | 3 | 7 | Low risk |
| United States | US-158 | Tiwari, et al. | 2021 | Cohort study | 3 | 1 | 2 | 6 | Moderate risk |
| United States | US-161 | Byrappagari, et al. | 2024 | Cohort study | 3 | 1 | 2 | 6 | Moderate risk |
| United States | US-162 | Bohnhoff, et al. | 2023 | Cohort study | 3 | 1 | 3 | 7 | Low risk |
| United States | US-169 | Pourat, et al. | 2025 | Cohort study | 2 | 1 | 3 | 6 | Moderate risk |
| United States | US-171 | Choi, et al. | 2025 | Cohort study | 3 | 1 | 3 | 7 | Low risk |
| United States | US-172 | Tuan, et al. | 2025 | Cohort study | 3 | 1 | 3 | 7 | Low risk |
| United States | US-177 | Khouja, et al. | 2022 | Cohort study | 3 | 1 | 3 | 7 | Low risk |
| United States | US-181 | Higgins, et al. | 2025 | Cohort study | 3 | 1 | 3 | 7 | Low risk |
| United States | US-182 | Mao, et al. | 2025 | Case-control study | 3 | 1 | 3 | 7 | Low risk |
| United States | US-187 | Peng, et al. | 2025 | Cohort study | 3 | 1 | 3 | 7 | Low risk |
| United States | US-189 | Schlotz, et al. | 2024 | Cohort study | 4 | 1 | 3 | 8 | Low risk |
| United States | US-190 | Bussard, et al. | 2024 | Cohort study | 3 | 1 | 3 | 7 | Low risk |
| United States | US-192 | Semprini, et al. | 2024 | Cohort study | 3 | 1 | 3 | 7 | Low risk |
| United States | US-193 | Hanna, et al. | 2024 | Cohort study | 3 | 0 | 1 | 4 | Moderate risk |
| United States | US-194 | Taylor, et al. | 2024 | Cohort study | 3 | 2 | 3 | 8 | Low risk |
| United States | US-196 | Tota, et al. | 2024 | Case-control study | 4 | 1 | 3 | 8 | Low risk |
| United States | US-199 | Michalowicz, et al. | 2023 | Cohort study | 3 | 1 | 2 | 6 | Moderate risk |
| United States | US-200 | Burns, et al. | 2023 | Cohort study | 3 | 1 | 3 | 7 | Low risk |
| United States | US-209 | Gordon, et al. | 2023 | mached cohort | 3 | 1 | 2 | 6 | Moderate risk |
| United States | US-211 | Peng, et al. | 2023 | Cohort study | 3 | 1 | 3 | 7 | Low risk |
| United States | US-214 | Brickhouse, et al. | 2023 | Cohort study | 4 | 1 | 3 | 8 | Low risk |
| United States | US-228 | Hubbard, et al. | 2022 | Cohort study | 3 | 1 | 3 | 7 | Low risk |
| United Kingdom | UK-2 | Wang et al. | 2012 | Retrospective cohort study | 4 | 2 | 2 | 8 | Low risk |
| United Kingdom | UK-3 | Burke & Lucarotti | 2012 | Retrospective cohort study | 4 | 1 | 2 | 7 | Low risk |
| United Kingdom | UK-4 | Fitzsimons et al. | 2013 | Retrospective population-based cohort study | 4 | 1 | 2 | 7 | Low risk |
| United Kingdom | UK-17 | Moore et al. | 2024 | Retrospective cohort study (natural experiment) | 3 | 2 | 2 | 7 | Low risk |
| United Kingdom | UK-23 | Moore et al. | 2024 | Retrospective cohort study (natural experiment) | 3 | 2 | 2 | 7 | Low risk |
| South Korea | KR-2 | Ha et al. | 2013 | Retrospective cohort study | 4 | 2 | 2 | 8 | Low risk |
| South Korea | KR-3 | Kwon et al. | 2015 | Nested case-control study within a national osteoporosis cohort | 4 | 2 | 2 | 8 | Low risk |
| South Korea | KR-4 | Lee et al. | 2016 | Nationwide population-based retrospective cohort study | 4 | 2 | 2 | 8 | Low risk |
| South Korea | KR-5 | Won et al. | 2017 | Population-based case-control study | 4 | 2 | 2 | 8 | Low risk |
| South Korea | KR-8 | Jung et al. | 2018 | Retrospective cohort study | 4 | 2 | 3 | 9 | Low risk |
| South Korea | KR-9 | Lee et al. | 2018 | Nationwide population-based retrospective cohort study | 4 | 2 | 2 | 8 | Low risk |
| South Korea | KR-13 | Seo et al. | 2020 | Nationwide population-based cohort study | 4 | 2 | 2 | 8 | Low risk |
| South Korea | KR-14 | Kang et al. | 2020 | Nationwide population-based retrospective cohort study | 4 | 2 | 2 | 8 | Low risk |
| South Korea | KR-15 | Chang et al. | 2021 | Nationwide population-based retrospective cohort study | 4 | 2 | 2 | 8 | Low risk |
| South Korea | KR-16 | Lee et al. | 2021 | Retrospective cohort study | 4 | 1 | 2 | 7 | Low risk |
| South Korea | KR-17 | Lee et al. | 2021 | Nationwide population-based cohort study | 4 | 2 | 2 | 8 | Low risk |
| South Korea | KR-18 | Lee et al. | 2021 | Retrospective cohort study | 4 | 2 | 2 | 8 | Low risk |
| South Korea | KR-H19 | Jae-Hong Lee et al. | 2015 | Nationwide retrospective cohort study | 4 | 2 | 2 | 8 | Low risk |
| South Korea | KR-H20 | Park et al. | 2019 | Nationwide population-based retrospective cohort study | 4 | 2 | 2 | 8 | Low risk |
| South Korea | KR-21 | Lee | 2024 | Nationwide retrospective cohort study | 4 | 2 | 2 | 8 | Low risk |
| South Korea | KR-23 | Son et al. | 2025 | Nationwide population-based nested case–control study | 4 | 2 | 2 | 8 | Low risk |
| South Korea | KR-24 | Park et al. | 2025 | Nationwide population-based cohort study | 4 | 2 | 2 | 8 | Low risk |
| South Korea | KR-25 | Lee & Kim | 2025 | Nationwide retrospective cohort study | 4 | 2 | 2 | 8 | Low risk |
| South Korea | KR-26 | Park et al. | 2024 | Nationwide retrospective cohort study | 4 | 2 | 2 | 8 | Low risk |
| South Korea | KR-28 | Kim et al. | 2025 | Longitudinal population-based cohort study | 4 | 2 | 2 | 8 | Low risk |
| South Korea | KR-31 | Cho et al. | 2024 | Population-based retrospective cohort study | 4 | 2 | 2 | 8 | Low risk |
| South Korea | KR-32 | Kwak et al. | 2023 | Nationwide population-based cohort study | 4 | 2 | 2 | 8 | Low risk |
| South Korea | KR-33 | Park et al. | 2023 | Nationwide population-based cohort study | 4 | 2 | 2 | 8 | Low risk |
| South Korea | KR-34 | Park et al. | 2023 | Retrospective cohort study | 4 | 2 | 2 | 8 | Low risk |
| South Korea | KR-35 | Han et al. | 2023 | Nationwide matched cohort study | 4 | 2 | 2 | 8 | Low risk |
| South Korea | KR-36 | Park et al. | 2023 | Nationwide longitudinal cohort study | 4 | 2 | 2 | 8 | Low risk |
| South Korea | KR-37 | Park et al. | 2023 | Nationwide cohort study | 4 | 2 | 2 | 8 | Low risk |
| South Korea | KR-38 | Kwoen et al. | 2023 | National cohort-based study | 4 | 2 | 2 | 8 | Low risk |
| South Korea | KR-39 | Ahn & Kim | 2022 | Retrospective cohort study | 3 | 1 | 2 | 6 | Moderate risk |
| South Korea | KR-40 | Song et al. | 2023 | Population-based retrospective cohort with machine-learning analysis | 4 | 0 | 2 | 6 | Moderate risk |
| Taiwan | TW-2 | Liao et al. | 2011 | Population-based retrospective cohort study | 4 | 2 | 2 | 8 | Low risk |
| Taiwan | TW-3 | Su et al. | 2012 | Nationwide population-based cohort study | 4 | 2 | 2 | 8 | Low risk |
| Taiwan | TW-4 | Lian et al. | 2013 | Retrospective cohort study | 3 | 1 | 2 | 6 | Moderate risk |
| Taiwan | TW-5 | Wu et al. | 2013 | Population-based case-control study | 4 | 2 | 2 | 8 | Low risk |
| Taiwan | TW-6 | Keller et al. | 2013 | Population-based case-control study | 4 | 2 | 2 | 8 | Low risk |
| Taiwan | TW-7 | Chen et al. | 2013 | Nationwide population-based case-control study | 4 | 2 | 2 | 8 | Low risk |
| Taiwan | TW-9 | Chen et al. | 2013 | Nationwide population-based retrospective cohort study | 4 | 2 | 2 | 8 | Low risk |
| Taiwan | TW-10 | Tseng | 2014 | Nationwide retrospective cohort study | 4 | 2 | 2 | 8 | Low risk |
| Taiwan | TW-14 | Lee et al. | 2014 | Retrospective population-based cohort study | 4 | 2 | 2 | 8 | Low risk |
| Taiwan | TW-15 | Chen et al. | 2014 | Population-based retrospective cohort study | 4 | 2 | 2 | 8 | Low risk |
| Taiwan | TW-16 | Lee et al. | 2014 | Nationwide population-based retrospective cohort study | 4 | 2 | 2 | 8 | Low risk |
| Taiwan | TW-17 | Chang et al. | 2014 | Population-based 5-year follow-up cohort study | 4 | 2 | 2 | 8 | Low risk |
| Taiwan | TW-20 | Lee et al. | 2015 | Nationwide population-based retrospective cohort study | 4 | 2 | 2 | 8 | Low risk |
| Taiwan | TW-22 | Ko et al. | 2015 | Nationwide population-based cohort study | 4 | 2 | 2 | 8 | Low risk |
| Taiwan | TW-23 | Shen et al. | 2015 | Nationwide population-based cohort study | 4 | 2 | 2 | 8 | Low risk |
| Taiwan | TW-24 | Tsai et al. | 2015 | Nationwide retrospective cohort study | 4 | 2 | 2 | 8 | Low risk |
| Taiwan | TW-26 | Wu et al. | 2016 | Population-based case-control study | 4 | 2 | 3 | 9 | Low risk |
| Taiwan | TW-28 | Lin et al. | 2016 | Population-based retrospective cohort study | 4 | 2 | 2 | 8 | Low risk |
| Taiwan | TW-30 | Hung et al. | 2016 | Population-based case-control study | 4 | 2 | 3 | 9 | Low risk |
| Taiwan | TW-31 | Chang et al. | 2016 | Population-based cohort study | 4 | 2 | 2 | 8 | Low risk |
| Taiwan | TW-32 | Hsieh et al. | 2016 | Population-based retrospective cohort study | 4 | 2 | 3 | 9 | Low risk |
| Taiwan | TW-33 | Hung et al. | 2016 | Population-based matched case-control study | 4 | 2 | 3 | 9 | Low risk |
| Taiwan | TW-36 | Shen et al. | 2016 | Propensity-matched retrospective cohort study | 4 | 2 | 2 | 8 | Low risk |
| Taiwan | TW-37 | Shen et al. | 2017 | Nationwide population-based retrospective cohort study | 4 | 2 | 2 | 8 | Low risk |
| Taiwan | TW-38 | Chang et al. | 2017 | Population-based retrospective cohort study | 4 | 2 | 2 | 8 | Low risk |
| Taiwan | TW-39 | Mau et al. | 2017 | Population-based cohort study | 4 | 2 | 2 | 8 | Low risk |
| Taiwan | TW-40 | Lin et al. | 2017 | Nationwide population-based case-control study | 4 | 2 | 3 | 9 | Low risk |
| Taiwan | TW-41 | Wu et al. | 2017 | Nationwide population-based case-control study | 4 | 2 | 3 | 9 | Low risk |
| Taiwan | TW-42 | Lin et al. | 2017 | Population-based matched case-control study | 4 | 2 | 2 | 8 | Low risk |
| Taiwan | TW-43 | Shen et al. | 2017 | Propensity-matched cohort study | 4 | 2 | 2 | 8 | Low risk |
| Taiwan | TW-44 | Tsai et al. | 2017 | Nationwide cohort study | 4 | 2 | 2 | 8 | Low risk |
| Taiwan | TW-45 | Chuang et al. | 2018 | Retrospective cohort study | 4 | 2 | 2 | 8 | Low risk |
| Taiwan | TW-47 | Yu et al. | 2018 | Nationwide population-based case-control study | 4 | 2 | 3 | 9 | Low risk |
| Taiwan | TW-48 | Chi et al. | 2018 | Population-based matched cohort study | 4 | 2 | 2 | 8 | Low risk |
| Taiwan | TW-49 | Lin et al. | 2018 | Nationwide population-based cohort study | 4 | 2 | 2 | 8 | Low risk |
| Taiwan | TW-50 | Hung et al. | 2018 | Retrospective cohort study | 4 | 2 | 2 | 8 | Low risk |
| Taiwan | TW-51 | Wang et al. | 2018 | Nationwide population-based retrospective cohort study | 4 | 2 | 2 | 8 | Low risk |
| Taiwan | TW-52 | Hung et al. | 2019 | Population-based case-control study | 4 | 2 | 3 | 9 | Low risk |
| Taiwan | TW-53 | Huang et al. | 2019 | Nationwide matched case-control study nested within HNC cohort | 4 | 2 | 3 | 9 | Low risk |
| Taiwan | TW-54 | Teng et al. | 2019 | Nationwide population-based retrospective cohort study | 4 | 2 | 2 | 8 | Low risk |
| Taiwan | TW-55 | Huang et al. | 2019 | Nationwide population-based retrospective cohort study | 4 | 2 | 2 | 8 | Low risk |
| Taiwan | TW-56 | Tong et al. | 2019 | Cohort study | 4 | 2 | 2 | 8 | Low risk |
| Taiwan | TW-59 | Chang et al. | 2020 | Nationwide population-based cohort study | 4 | 2 | 2 | 8 | Low risk |
| Taiwan | TW-60 | Lin et al. | 2020 | Nationwide nested case-control study | 4 | 2 | 3 | 9 | Low risk |
| Taiwan | TW-61 | Huang et al. | 2020 | Nationwide population-based retrospective cohort study | 4 | 2 | 2 | 8 | Low risk |
| Taiwan | TW-62 | Chen et al. | 2020 | Retrospective population-based cohort study | 4 | 2 | 2 | 8 | Low risk |
| Taiwan | TW-63 | Tsai et al. | 2020 | Population-based matched case-control study | 4 | 2 | 3 | 9 | Low risk |
| Taiwan | TW-64 | Chen et al. | 2021 | Nested case-control study | 4 | 2 | 3 | 9 | Low risk |
| Taiwan | TW-65 | Chuang et al. | 2020 | Population-based retrospective cohort study | 4 | 2 | 2 | 8 | Low risk |
| Taiwan | TW-66 | Lee et al. | 2020 | Nationwide retrospective cohort study | 4 | 2 | 2 | 8 | Low risk |
| Taiwan | TW-68 | Sun et al. | 2020 | Population-based retrospective cohort study | 4 | 2 | 2 | 8 | Low risk |
| Taiwan | TW-69 | Huang et al. | 2021 | Population-based cohort study (bidirectional analysis) | 4 | 2 | 2 | 8 | Low risk |
| Taiwan | TW-71 | Chen et al. | 2021 | Nationwide retrospective cohort study | 4 | 2 | 2 | 8 | Low risk |
| Taiwan | TW-72 | Sia et al. | 2021 | Nationwide retrospective cohort study | 4 | 2 | 2 | 8 | Low risk |
| Taiwan | TW-73 | Lin et al. | 2021 | Nationwide population-based case-control study | 4 | 2 | 2 | 8 | Low risk |
| Taiwan | TW-74 | Li et al. | 2021 | Nationwide retrospective cohort study | 3 | 2 | 2 | 7 | Low risk |
| Taiwan | TW-76 | Chang et al. | 2022 | Population-based cohort study | 4 | 2 | 2 | 8 | Low risk |
| Taiwan | TW-77 | Fu et al. | 2022 | Population-based matched cohort study | 4 | 2 | 2 | 8 | Low risk |
| Taiwan | TW-78 | Huang et al. | 2022 | Population-based case-control study (cross-sectional case-control design) | 4 | 2 | 2 | 8 | Low risk |
| Taiwan | TW-79 | Chiang et al. | 2025 | Retrospective cohort study | 4 | 2 | 3 | 9 | Low risk |
| Taiwan | TW-80 | Yeh et al. | 2025 | Population-based nested case-control study | 4 | 2 | 3 | 9 | Low risk |
| Taiwan | TW-81 | Juan et al. | 2025 | Cohort-based nested case-control study | 4 | 2 | 3 | 9 | Low risk |
| Taiwan | TW-82 | Yang et al. | 2025 | Population-based case-control study | 4 | 2 | 3 | 9 | Low risk |
| Taiwan | TW-83 | Yang et al. | 2025 | Retrospective population-based cohort study | 4 | 2 | 2 | 8 | Low risk |
| Taiwan | TW-84 | Yang et al. | 2025 | Population-based case-control study | 4 | 2 | 3 | 9 | Low risk |
| Taiwan | TW-85 | Liu et al. | 2024 | Nationwide population-based nested case-control study | 4 | 2 | 2 | 8 | Low risk |
| Taiwan | TW-86 | Ono et al. | 2024 | Retrospective cohort study | 4 | 2 | 2 | 8 | Low risk |
| Taiwan | TW-88 | Sung et al. | 2023 | Retrospective cohort study | 4 | 2 | 2 | 8 | Low risk |
| Taiwan | TW-89 | Liou et al. | 2023 | Retrospective population-based cohort study | 4 | 2 | 2 | 8 | Low risk |
| Taiwan | TW-90 | Liao et al. | 2023 | Retrospective population-based nested case-control study | 4 | 2 | 3 | 9 | Low risk |
| Taiwan | TW-91 | Juan et al. | 2022 | Secondary population-based cohort study | 4 | 2 | 2 | 8 | Low risk |
| Taiwan | TW-92 | Tseng | 2022 | Retrospective cohort study | 4 | 2 | 2 | 8 | Low risk |
| Taiwan | TW-93 | Yeh et al. | 2022 | Nationwide population-based cohort study | 4 | 2 | 2 | 8 | Low risk |
| Taiwan | TW-94 | Chen et al. | 2022 | Population-based nested case-control study | 4 | 2 | 3 | 9 | Low risk |
| Germany | DE-2 | Raedel et al. | 2015 | Cohort study | 2 | 0 | 2 | 4 | Moderate risk |
| Germany | DE-3 | Raedel et al. | 2015 | Cohort study | 2 | 0 | 2 | 4 | Moderate risk |
| Germany | DE-4 | Raedel et al. | 2016 | Cohort study | 2 | 0 | 2 | 4 | Moderate risk |
| Germany | DE-7 | Raedel et al. | 2017 | Cohort study | 2 | 1 | 2 | 5 | Moderate risk |
| Germany | DE-8 | Raedel et al. | 2017 | Cohort study | 2 | 0 | 2 | 4 | Moderate risk |
| Germany | DE-9 | Raedel et al. | 2019 | Cohort study | 3 | 1 | 2 | 6 | Moderate risk |
| Germany | DE-11 | Hempel et al. | 2020 | Cohort study | 2 | 1 | 2 | 5 | Moderate risk |
| Germany | DE-12 | Aarabi et al. | 2020 | Cohort study | 3 | 1 | 1 | 5 | Moderate risk |
| Germany | DE-13 | Raedel et al. | 2021 | Cohort study | 2 | 0 | 2 | 4 | Moderate risk |
| Germany | DE-14 | Blaschke et al. | 2021 | Cohort study | 2 | 1 | 3 | 6 | Moderate risk |
| Germany | DE-15 | Schwendicke et al. | 2021 | Cohort study | 2 | 1 | 2 | 5 | Moderate risk |
| Germany | DE-17 | Henken et al. | 2025 | Cohort study | 4 | 1 | 3 | 8 | Low risk |
| Germany | DE-18 | Schweyen et al. | 2025 | Cohort study | 3 | 1 | 2 | 6 | Moderate risk |
| Germany | DE-20 | Raedel et al. | 2024 | Cohort study | 4 | 0 | 2 | 6 | Moderate risk |
| Germany | DE-23 | Raedel et al. | 2022 | 2-3. Case-control | 3 | 0 | 3 | 6 | Moderate risk |
| Germany | DE-24 | Czwikla et al. | 2022 | Cohort study | 3 | 1 | 3 | 7 | Low risk |
| Germany | DE-25 | Raedel et al. | 2022 | Cohort study | 4 | 0 | 2 | 6 | Moderate risk |
| Netherlands | NL-4 | Hummel, et al. | 2021 | Cohort study | 4 | 1 | 3 | 8 | Low risk |
| Netherlands | NL-5H | Smits, et ak. | 2020 | Cohort study | 3 | 1 | 3 | 7 | Low risk |
| Netherlands | NL-9 | Beukers, e al. | 2023 | Cohort study | 3 | 1 | 3 | 7 | Low risk |
| France | FR-2 | Tubiana, et al. | 2017 | Cohort study | 4 | 1 | 3 | 8 | Low risk |
| France | FR-6 | Blotiere, et al. | 2019 | Cohort study | 4 | 1 | 3 | 8 | Low risk |
| France | FR-14 | Goulabchand, et al. | 2022 | Cohort study | 4 | 1 | 2 | 7 | Low risk |
| Denmark | DK-3 | Iversen, et al. | 2021 | Cohort study | 2 | 1 | 3 | 6 | Moderate risk |
| Australia | AU-7 | Stormon, et al. | 2025 | 2-2.cross-sectional | 4 | 1 | 2 | 7 | Low risk |
| Canada | CA-3 | Singhal, et al | 2016 | Cohort study | 4 | 1 | 3 | 8 | Low risk |
| Canada | CA-4 | Malic, et al. | 2020 | Cohort study | 4 | 1 | 3 | 8 | Low risk |
| Canada | CA-6 | Sawani, et al | 2025 | Cohort study | 3 | 1 | 3 | 7 | Low risk |
| Canada | CA-7 | Yazdanbakhsh, et al. | 2025 | Cohort study | 4 | 1 | 2 | 7 | Low risk |
| Canada | CA-8 | MiriMoghaddam, et al. | 2024 | Cohort study | 4 | 1 | 3 | 8 | Low risk |
| Canada | CA-9 | MiriMoghaddam, et al. | 2024 | Cohort study | 4 | 1 | 3 | 8 | Low risk |
| Canada | CA-10 | Malic, et al. | 2023 | Cohort study | 4 | 1 | 3 | 8 | Low risk |
| Canada | CA-11 | Yazdanbakhsh, et al. | 2024 | Cohort study | 3 | 1 | 2 | 6 | Moderate risk |
| Switzerland | CH-1 | Laager, et al. | 2024 | Cohort study | 4 | 1 | 2 | 7 | Low risk |
| New Zealand | NZ-1 | Schluter, et al. | 2025 | Cohort study | 3 | 1 | 3 | 7 | Low risk |

**Note:** Only studies eligible for NOS assessment are shown. The overall risk of bias was classified as low for total NOS scores of ≥7, moderate for scores of 4–6, and high for scores of ≤3.

NOS, Newcastle–Ottawa Scale.

**Supplementary Appendix 1**

**Standardized prompt used to generate preliminary Newcastle-Ottawa Scale (NOS) ratings**

| **Model/service** | ChatGPT, Model GPT-5.5 Thinking (OpenAI, San Francisco, CA, USA) |
| --- | --- |
| **Custom GPT** | Newcastle-Ottawa Quality Assessment Scale (NOS) |
| **URL** | https://chatgpt.com/g/g-Xr35IAPKg-newcastle-ottawa-quality-assessment-scale-nos |
| **Access period** | June 2026 to July 2026 |
| **Materials supplied** | Full text of each article eligible for NOS appraisal |
| **Intended role** | First-pass, item-level draft for verification by a human reviewer |

For each eligible cohort or case-control study, the article full text was supplied to the model together with the standardized prompt below. The prompt is reproduced verbatim.

# **Verbatim prompt**

Please assess this cohort/case-control study using the Newcastle–Ottawa Scale.

**Important rules:**

- Do not infer information that is not explicitly stated.

- If the information is unclear, mark it as unclear.

- For each NOS item, provide the judgment, provisional star, and supporting text or section.

- This is a preliminary assessment only; final judgments will be made by a human reviewer.

- Please summarize the results as Selection, Comparability, Outcome/Exposure, and Total NOS score.

# **Human verification and final decision**

All model-generated item ratings were treated as provisional. One reviewer checked every item rating and its supporting text against the full article and revised any judgment not supported by the source. Unclear or borderline cases were referred to a second reviewer. Final item ratings, domain scores, total NOS scores, and risk classifications were reviewer-determined; no model output was accepted as a final judgment.
